# Self-reported perception of statistical literacy: Evidence from a National Survey of U.S. Adults

**DOI:** 10.1371/journal.pone.0350282

**Published:** 2026-06-24

**Authors:** Samuel Anyaso-Samuel, Mark Louie Ramos

**Affiliations:** 1 Biostatistics Branch, Division of Cancer Epidemiology and Genetics, National Cancer Institute,‌‌ Bethesda, Maryland, United States of America; 2 Department of Health Policy and Administration, The Pennsylvania State University, ‌‌University Park, Pennsylvania, United States of America; University of Potsdam: Universitat Potsdam, GERMANY

## Abstract

Amid growing recognition of the role statistical literacy plays in informed decision-making, we examined self-reported statistical literacy and willingness to apply statistical reasoning in everyday contexts using data if they understood statistics better through secondary data analysis of a nationally representative survey of U.S. adults. The survey showed that 62% of U.S. adults self-report having little to no idea what statistics or statistical concepts like p-values are, but 90% of them would base decisions on reported statistics at least sometimes if they understood them better. Survey-weighted ordinal regression revealed that higher educational attainment was positively associated with perceived statistical literacy, while older generational cohorts reported lower levels. Individuals who rated themselves as more statistically literate expressed greater willingness to use statistical information provided they understood it better. These findings highlight the urgent need to further expand statistical education across both formal curricula and informal learning environments to empower public engagement with data-driven issues.

## Introduction

With the United States’ present focus on developing literacy in Artificial Intelligence and Large Language Models (LLMs) [1–3], it is important to point out that a critical component of AI literacy is statistical literacy. The algorithms that underpin machine learning and generative AI systems are built on probabilistic models, statistical inference, and uncertainty quantification [4–6]. The ability to understand these statistical underpinnings is vital for both interpreting AI-driven outputs and making informed judgments about their reliability, fairness, and societal impact [7]. Without statistical literacy, individuals risk becoming passive consumers of AI-generated information rather than critical evaluators. More broadly, statistical literacy equips the public to interpret and critique evidence-based claims across domains of daily life and policy. For example, public debates about associations between prenatal acetaminophen use and autism highlight the importance of recognizing the difference between correlation and causation and how evidence of causation in light of observed association is attempted using statistical tools [8]. In line with this, we sought to explore perceptions of statistical literacy among adults in the U.S.

Statistical literacy is defined as the ability to interpret, critically evaluate, and communicate statistical information involving stochastic phenomena [9]. Despite the central role of statistics in modern data-driven society, widespread challenges remain in fostering meaningful understanding. While statistics has been part of the U.S. K-12 curriculum for decades and has been the subject of various rounds of development and reform [10–12], developing literacy in the practical use of statistical tools, such as understanding confidence intervals or p-values, has remained challenging even at the higher education level [9,13–17]. These challenges become particularly pressing in an age when probabilistic reasoning underlies critical domains such as health decision-making, risk communication, and public trust in science [18,19]

Attempts to measure statistical literacy at the national level have historically been limited. Yet, the need for a nuanced understanding of statistical reasoning has never been greater, particularly as the public increasingly encounters statistical information through AI systems, health communication, financial decisions, and evidence-based policymaking [20]. The National Science Foundation’s *Science and Engineering Indicators* included embedded measures of probability and scientific reasoning, reporting in 2018 that only about 43% of Americans could correctly answer basic questions about probability and scientific experiments [21]. Broader national surveys tend to report numerical or quantitative literacy more generally, without specific focus on statistical literacy [22,23]. In particular, results from the latest Program for the International Assessment of Adult Competencies (PIAAC) study found that adult numeracy in the U.S. fell between 2017 and 2023 and that only 38% of adults had level 3 or better numeracy, with only 10% at level 4 and 2% at level 5 [24]. This is relevant because level 3 is the first level where a component of statistical reasoning is mentioned (i.e., “(able to) evaluate claims and stated relationships using a variety of data sources”), and statistical literacy is explicitly mentioned as among other components of level 4 and 5: “(able to) reflect and reason mathematically to review and evaluate the validity of statistical or mathematical conclusions, claims or arguments” (level 4) and “(able to) demonstrate an understanding of statistical concepts and can critically reflect on whether a data set can be used to support or refute a claim” (level 5) [25]. However, each level has multiple components and so cannot be considered as measuring statistical literacy specifically.

At the school level, evidence from international and national assessments similarly points to uneven preparation in statistics and data-related competencies. The Programme for International Student Assessment (PISA) 2022 introduced an uncertainty and data subscale within mathematics, revealing that many U.S. students struggle with interpreting data, reasoning under uncertainty, and making evidence-based judgments [26]. Among K-12 students in the U.S. who participated in the PISA 2022, 66% scored at level 2 or better of 6 levels for mathematical literacy [26]. They scored consistently across each subscale, including the uncertainty and data subscale that is most related to statistical literacy. However, level interpretations are for the overall score, with components related to data literacy appearing as early as level 2 (“They can extract relevant information from one or more sources that use slightly more complex modes of representation, such as two-way tables, charts”) and components related to decision making under uncertainty appearing gradually at higher levels [27]. National results from the National Assessment of Educational Progress (NAEP) further show that while exposure to data and statistics has increased, student proficiency remains limited and unevenly distributed [28]. Together, these findings suggest that both K–12 education and adult learning systems leave many individuals underprepared for engaging critically with statistical information.

Moreover, an important element not covered by these attempts at measuring statistical literacy at the national level is subjective numeracy, which is defined as a person’s self‑assessment of their quantitative ability and their comfort or preference for using numerical information [29]. Subjective numeracy has been shown as highly associated with objective numeracy both for mathematics in general [30] and for statistical literacy in particular [31]. More than this, subjective numeracy captures motivational factors such as numerical self-efficacy and math anxiety that influence whether individuals engage with quantitative information in the first place [32]. People who believe themselves to understand mathematics better are more willing to engage with quantitative information and rely on numerical information in judgments and decisions.[33] While there are numerous papers that attempt to examine self-perceptions of statistical literacy in the U.S. [34–36], these have mainly focused on students in higher education rather than the general population. As such, this paper contributes to the literature by presenting results from a nationally representative survey of U.S. adults that explores self-reported perception of statistical literacy and individuals’ willingness to engage with statistical concepts in decision-making. Specifically, it estimates the distribution of U.S. adults based on (a) their perception of their own understanding of statistical concepts, and (b) their willingness to use statistical reasoning in making personal and societal decisions if they understood statistical concepts better. This “conditional willingness” to use statistical reasoning is aligned with what is known about subjective numeracy in general [32,33], but is sought to be measured in the context of subjective perception of statistical literacy in particular. We also explored the association of these constructs with different demographic variables in order to characterize their distributions with respect to the U.S. population further.

## Methodology

### Sample

Data for this study was collected by Verasight, a market research and survey provider specializing in producing high-quality, policy-relevant public opinion data. Verasight crowdsourced questions for a multi-topic survey from attendees of the 2025 Joint Statistical Meetings that happened from August 2–7, 2025 in Nashville, TN. During this event, we submitted two questions.

Verasight conducted the survey between August 13 and August 18, 2025, with a nationally representative final sample of 1,000 U.S. adult residents. Approximately 17,153 Verasight Community members, composed of individuals recruited via random address-based sampling, random person-to-person text messaging, and dynamic online targeting, were invited to participate in the survey, of whom 6.73% responded. All community members underwent multi‑step authentication, including SMS verification using a mobile phone registered with a major U.S. carrier and within‑survey technologies such as Google reCAPTCHA v3 to confirm the absence of non‑human responses. Verasight also conducted extensive post‑fieldwork quality checks, including verification of U.S. IP addresses, detection of duplicate respondents, removal of suspected non‑human responses, and exclusion of respondents who failed attention checks, exhibited straight‑lining behavior, or completed the survey in less than 30% of the median completion time. After all quality‑assurance procedures, the final analytic sample consisted of 1,000 respondents, representing 5.83% of those invited. Verasight Community members received points redeemable for PayPal or Venmo payments, gift cards, or charitable donations, and respondents were never routed between surveys; compensation was provided for each invited survey, eliminating incentives to misrepresent eligibility.

The data were weighted to match the July 2025 Current Population Survey on age, race/ethnicity, sex, income, education, region, and metropolitan status, as well as to a running three-year average of partisanship distributions from the Pew Research Center NPORS benchmarking surveys, and population benchmarks of 2024 vote. Weights were calculated using iterative proportional fitting to adjust the sample characteristics to match population marginals on the listed demographic variables. The margin of sampling error for the full sample, which accounts for the design effect and was calculated using the classical random sampling formula, was + /- 3.1% at the 95% confidence level.

### Design

This study is a secondary data analysis of results from specific questions asked in the Verasight survey. After they conducted the survey, Verasight provided us with access to de‑identified responses. We explored responses specific or related to the two questions we had contributed.

### Measures

We were interested in two measures reflected by the questions we contributed to the survey. The first question was: “How much do you understand about statistics and p-values?” This was a multiple choice question with possible responses following this four-point scale: (1) None – I have no idea what any of these are, (2) Little - I’ve heard of them or read about them, (3) Not much – but I learned them in school, and (4) Very much – I use them regularly. The categories were designed to capture different levels of perceived statistical familiarity, with (1) to (2) indicating minimal or no formal exposure with statistical concepts, (3) indicating limited formal exposure, such as through K-12 statistics content or undergraduate introductory course content, and (4) indicating active or professional engagement with statistics. Naturally, this is just perception of statistical literacy and not a measurement of actual literacy, but we contend that it is at least a meaningful indicator of how confident respondents feel engaging with statistical concepts. The second question was “How often would you base decisions on reported statistics if you understood it better?” Possible responses to this question were: (1) Never, (2) Sometimes, (3) Often, (4) Always. This question is conceptually intertwined with the first question, and we expect there to be some association just from the general reasoning that the greater one’s perception is about how well they understand something, the more likely they might be inclined to use it, but this relationship is not necessarily deterministic so we were interested in how large the association is and how the association might change when including covariates. In addition to these questions, we also had access to responses for all the other questions asked in the survey, including questions about demographics and software use. A copy of the original questionnaire is included as supplementary material in S1 File.

### Procedure

All respondents were recruited and provided consent via email from the Verasight Community. Respondents received a link to the online survey and were informed that the survey was optional and that they may exit at any time. Respondents then proceeded to answer the survey questions, including the two that we contributed, in no particular order. Once finished, respondents submitted their responses. The median length of time to complete the survey was 4.68 minutes.

All personally identifiable information was removed before Verasight shared the survey data with us and other external question contributors. Among respondents, 68% completed the survey on mobile devices or tablets, and 32% on desktops.

### Analysis

We used survey-weighted ordinal logistic regression models (cumulative logit) to analyze survey responses to our two outcome variables. All analyses incorporated survey weights to adjust for unequal probabilities of selection and post-stratification and provide population-representative estimates. We included covariates representing educational attainment, age, income, gender, political affiliation, and race ethnicity. Model fit was assessed using standard diagnostics for ordinal logistic regression, including checks for the proportional odds assumption. Analyses were conducted using R version 4.5.1, with the survey package [37] for complex survey design adjustments.

## Results and discussion

### Population characteristics

The analytic sample of 1,000 U.S. adults reflected broad demographic diversity that is consistent with expectation for a U.S. population representative sample (Table 1). Educational attainment was distributed across the spectrum, with 37% (95% CI: 34–40) reporting a high school diploma or less, 23% (20–25) holding a bachelor’s degree, and 15% (13–17) holding a postgraduate degree. Household income was also varied, with 27% (24–29) reporting incomes under $50,000, while 29% (26–31) fell between $50,000 and $74,999, and about 26% reported incomes above $100,000. Political affiliation was evenly distributed across Democrats (29.9%), Republicans (30.6%), and Independents (29.5%), with 10% reporting another or no affiliation. The racial and ethnic composition included 60% (57–63) non-Hispanic White, 13% (11–15) Black, and 11% (9–13) Hispanic White respondents, alongside smaller proportions of Asian, mixed race, Native American, and other groups. Gender was nearly balanced, with 53% identifying as female and 46% as male, and the age distribution spanned all major generations, with Baby Boomers (28%), Generation X (28%), and Millennials (26%) comprising the largest groups. We defined the generation intervals based on the National Center for Principled Leadership & Research Ethics [38].

**Table 1:** Demographic characteristics of U.S. adults in the study sample.

	Proportion^a,b^	95% CI^a,b^	Benchmark^c,d^
Education			
High School or less	0.37	[0.34, 0.40]	0.37
Some college, no degree	0.16	[0.14, 0.18]	0.15
2-year or associate degree	0.10	[0.08, 0.11]	0.11
4-year or bachelor degree	0.23	[0.20, 0.25]	0.23
Post-graduate degree	0.15	[0.13, 0.17]	0.14
Income			
Less than $50,000	0.27	[0.24, 0.29]	0.30
$50,000 to under $75,000	0.29	[0.26, 0.31]	0.15
$75,000 to under $100,000	0.19	[0.17, 0.22]	0.12
$100,000 to under $150,000	0.14	[0.12, 0.16]	0.15
$150,000 to under $200,000	0.06	[0.04, 0.07]	0.10
More than $200,000	0.06	[0.04, 0.07]	0.16
Political Affiliation			
Independent	0.30	[0.27, 0.32]	0.33
Democrat	0.30	[0.27, 0.33]	0.28
Republican	0.30	[0.28, 0.34]	0.31
Other or none	0.10	[0.08, 0.12]	0.08
Race/Ethnicity			
Asian or Asian-American	0.05	[0.04, 0.07]	0.07
Black or African-American	0.13	[0.11, 0.15]	0.14
Hispanic White	0.11	[0.09, 0.13]	0.14
Mixed race	0.05	[0.04, 0.07]	0.03
Native American or American Indian	0.02	[0.01, 0.03]	0.02
Non-Hispanic White	0.60	[0.57, 0.63]	0.58
Some other race	0.03	[0.02, 0.05]	0.02
Gender			
Female	0.53	[0.50, 0.56]	0.51
Male	0.46	[0.43, 0.49]	0.49
Other	0.01	[0.00, 0.01]	0.00
Age Group			
Silent Gen (Born <=1945)	0.01	[0.01, 0.02]	0.04
Boomers (Born 1946–1964)	0.28	[0.25, 0.31]	0.25
Gen X (Born 1965–1980)	0.28	[0.25, 0.31]	0.26
Millennials (Born 1981–1996)	0.26	[0.23, 0.29]	0.27
Gen Z (Born 1997–2012)	0.17	[0.15, 0.20]	0.18

a) Estimates are weighted to July 2025 Current Population Survey benchmarks on age, race/ethnicity, sex, income, education, region, and metropolitan status. Partisan distributions were further calibrated to Pew Research Center’s National Public Opinion Reference Survey (NPORS) and the 2024 U.S. presidential vote.

b) Sample size: N = 1,000 U.S. adults. Estimates are proportions with 95% confidence intervals shown in brackets.

c) U.S. Census Bureau, Population Division, 2025, https://www.census.gov/data/datasets/time-series/demo/popest/2020s-national-detail.html

d) Pew Research Center NPORS, 2025, https://www.pewresearch.org/politics/fact-sheet/party-affiliation-fact-sheet-npors/

### Main findings

This subsection describes the main results of the study, which are the nationally-representative survey measurements of self-reported perception of statistical literacy and willingness to use statistics for decision-making given better understanding of it.

Regarding self-reported perception of statistical literacy Table 2 shows that 25% (22–28) of adults reported no understanding of statistics or p-values, while 37% (34–40) indicated only limited familiarity. About 27% (24–30) reported some school-based exposure, and only 11% (9–13) described themselves as regular users of statistics. These findings suggest that a majority of U.S. adults perceive themselves as having little to no working knowledge of basic statistical concepts.

**Table 2:** Self-Reported Perception of Statistical Literacy and Willingness to Use Statistics.

	Proportion^a,b^	95% CI^a,b^
How much do you understand about statistics and p-values?		
None, I have no idea what any of these are.	0.25	[0.22, 0.28]
Little, I’ve heard of them or read about them.	0.37	[0.34, 0.40]
Not much, but I learned them in school.	0.27	[0.24, 0.30]
Very much, I use them regularly.	0.11	[0.09, 0.13]
How often would you base decisions on reported statistics if you understood it better?		
Never	0.10	[0.08, 0.12]
Sometimes	0.49	[0.46, 0.52]
Often	0.33	[0.30, 0.36]
Always	0.08	[0.07, 0.10]

a) Estimates are survey-weighted and adjusted to national demographic and partisan benchmarks as described for Table 1.

b) Sample size: N = 1,000 U.S. adults. Estimates are proportions with 95% confidence intervals shown in brackets.

When asked about their willingness to use statistical information in decision-making if they understood it better, nearly half (49%, 46–52) responded “sometimes,” and another 33% (30–36) reported “often.” A smaller group indicated they would “always” base decisions on statistics (8%, 7–10), while 10% (8–12) stated they would never do so. Overall, approximately 90% of adults expressed at least some conditional willingness to use statistics for decision-making if their understanding improved.

The association between the two constructs is shown in Table 3. There is a modest positive association between self-reported perception of statistical literacy and willingness to use statistics for decision making given better understanding (Survey Weighted Spearman Rho = 0.36). Among those who reported not knowing about statistics at all, 78% would use it at least sometimes if they understood it better. This rises to 92% among those who reported some statistical literacy and 96% among those who reported at least learning about statistics in school.

**Table 3:** Self-Reported Statistical Literacy by Willingness to Use Reported Statistics for Decision-making.

	How often would you base decisions on reported statistics if you understood it better?			
Raw weighted counts	Never	Sometimes	Often	Always
None, I have no idea what any of these are.	55.58	141.18	48.20	4.84
Little, I’ve heard of them or read about them.	29.45	204.50	109.16	27.79
Not much, but I learned them in school.	9.88	120.22	122.37	18.76
Very much, I use them regularly.	4.29	24.70	46.59	32.49
	How often would you base decisions on reported statistics if you understood it better?			
Proportions by row	Never	Sometimes	Often	Always
None, I have no idea what any of these are.	0.22	0.57	0.19	0.02
Little, I’ve heard of them or read about them.	0.08	0.55	0.29	0.07
Not much, but I learned them in school.	0.04	0.44	0.45	0.07
Very much, I use them regularly.	0.04	0.23	0.43	0.30

### Ordinal regression model for self-reported statistical literacy

This subsection explores the first main construct further by modeling it under some demographic covariates.

**Fig 1:**
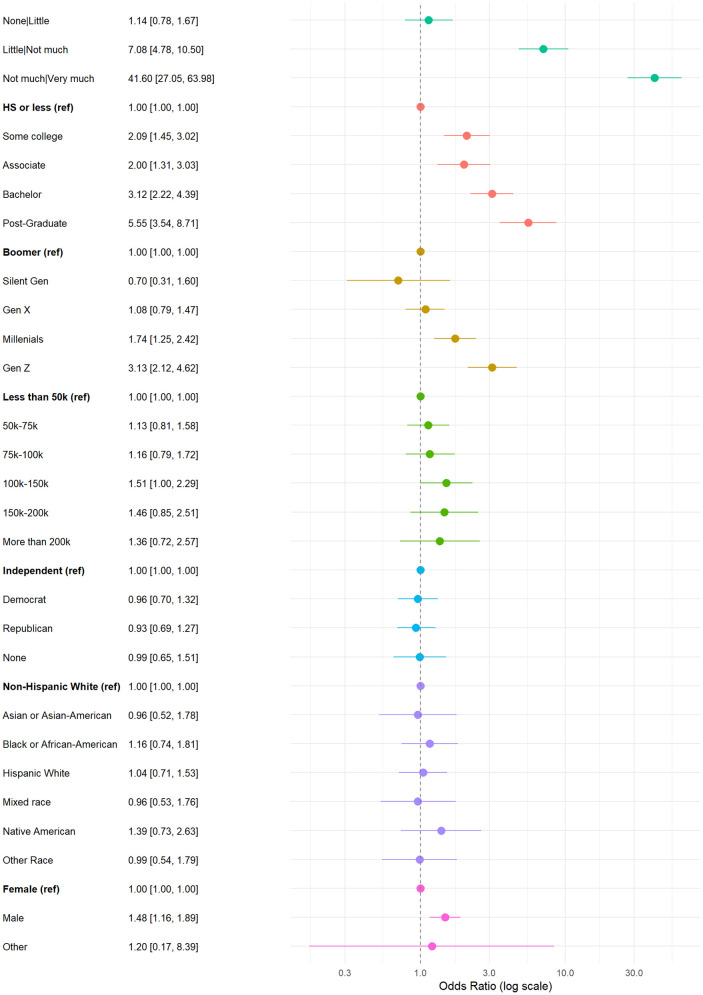
Ordinal regression that shows how each covariate is associated with reporting a higher level of perceived statistical literacy.

**Fig 2:**
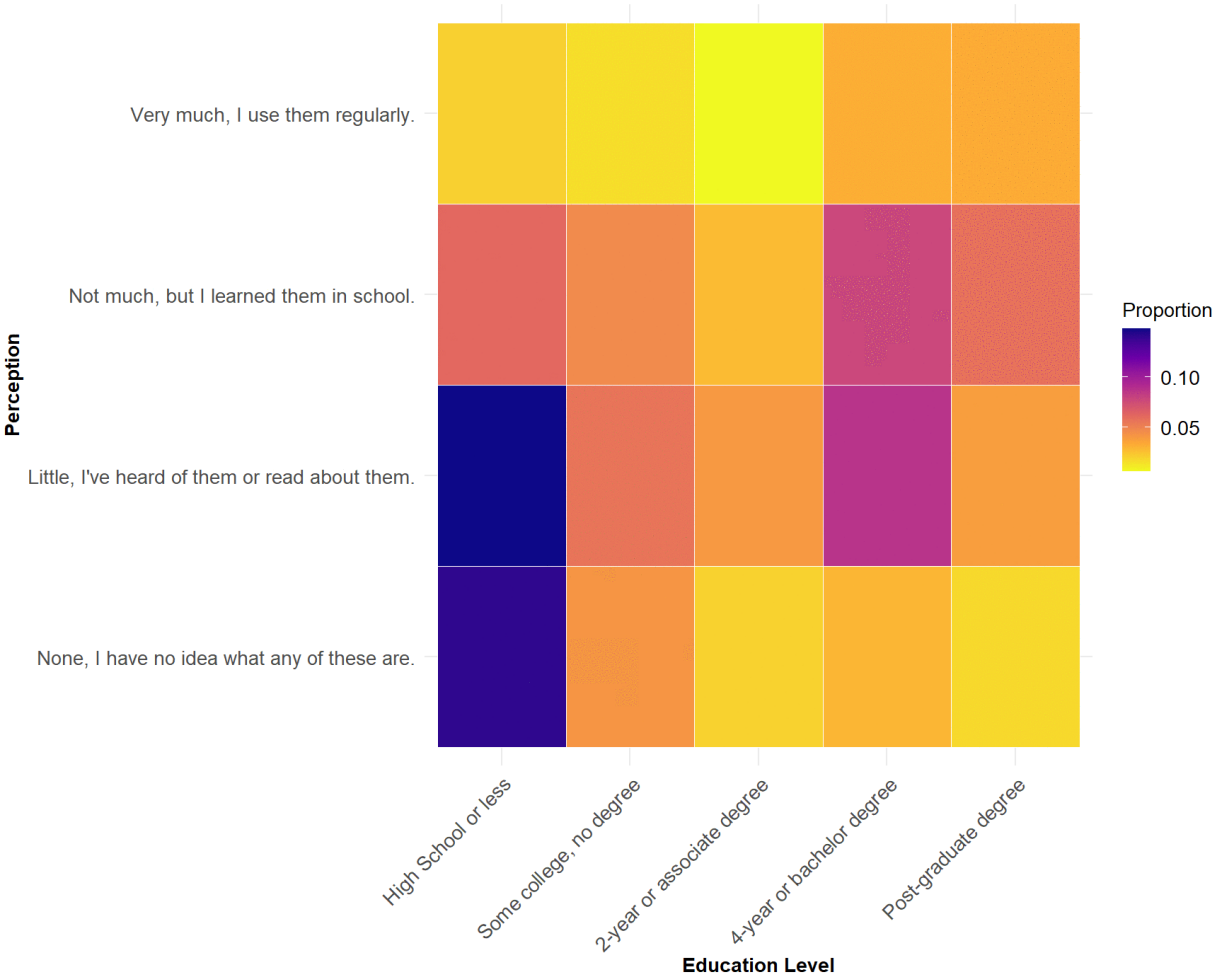
Self-reported Perception of Statistical Literacy by Education.

Fig 1 shows results for this ordinal regression model. The intercepts of the model show progressively higher baseline odds thresholds for each fixed ordinal level, indicating smaller differences in the latent trait between those who report “None” or “Little,” but much larger differences in the shift from “Little” to “Learned them in school” and even larger between “Learned them in school” and “Use them regularly.” Subsequent odds ratios for each covariate quantify the relative chance of reporting a higher level of perceived statistical literacy compared with that covariate’s reference category. Among demographic variables, education and age were found to be the strongest predictors of self-reported statistical literacy. For example, those with some college or an associate’s degree were found to be twice as likely to report higher levels of perceived statistical literacy than those with at most a high school education. Fig 2 examines this association further and shows that over 38% of people with at most a high school diploma reported that they “have no idea what statistics or p-values are,” compared to only 12% of those with a 4-year college degree or a postgraduate degree. On the other hand, reporting regular use of statistics is uncommon even among those with postgraduate education (22%), but still much higher than other education groups.

**Fig 3:**
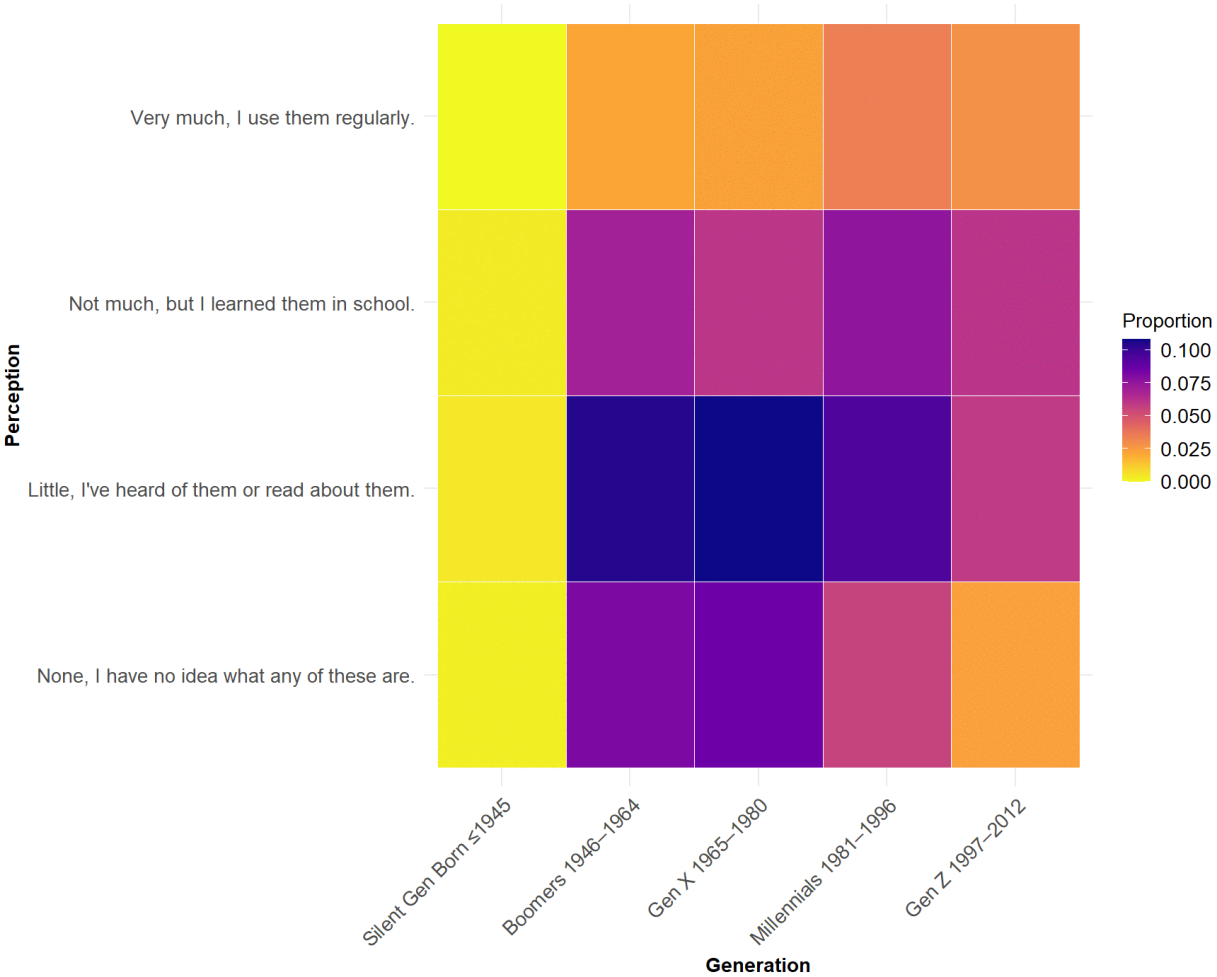
Self-reported Statistical Literacy by Generation.

The model also showed that younger generations have higher likelihood of self-reported perception of statistical literacy. Using Boomers as reference, later generations were found to have higher odds of reporting higher statistical literacy. Fig 3 shows that Gen Z, the youngest generation in the survey, had both the lowest proportion reporting no knowledge of statistics (14%) and the highest proportion reporting regular use (17%).

Males were also found to have slightly higher (OR [1.15,1.88]) self-reported perception of statistical literacy than females. None of the other demographic variables (income, political affiliation, race/ethnicity) were found to have statistically significant association with self-reported statistical literacy.

### Ordinal regression model for willingness to use statistics in decision-making

**Fig 4:**
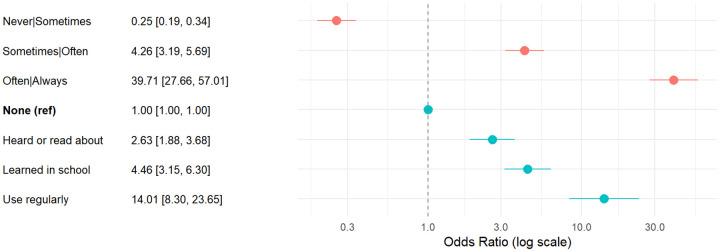
Ordinal regression estimating how self‑reported statistical literacy is associated with being more willing to use statistics in decision‑making.

**Fig 5:**
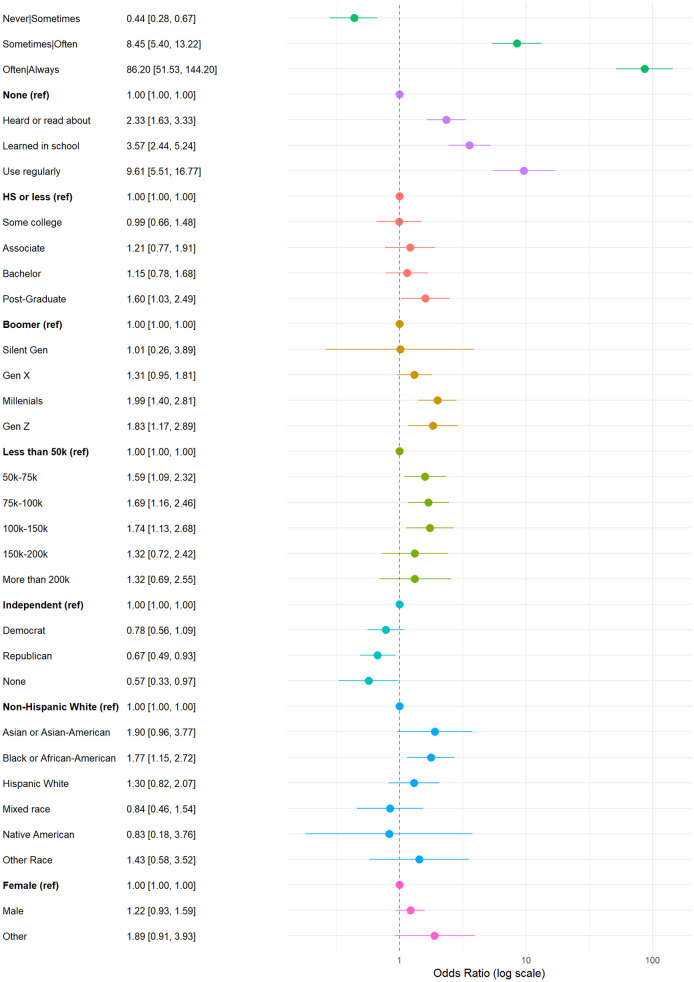
Ordinal regression estimating how self‑reported statistical literacy and some demographic variables are associated with being more willing to use statistics in decision‑making.

This subsection explores ordinal regression modeling for the second construct as the outcome. Fig 4 and Fig 5 show the odds ratios for modelling willingness to make decisions based on statistics, conditional on having better understanding about it. The intercepts in both models show that respondents were significantly more likely to report that they would “sometimes” rather than “never” use statistics in decision-making. In the model with only self-reported statistical literacy (Fig 2), higher levels of perceived understanding were strongly and consistently associated with greater willingness. This relationship persisted after adjusting for demographic covariates (Fig 3), though the magnitude of the effects was somewhat attenuated.

There is positive but weak association between willingness to use statistics for decision-making and education or generation, though this is at least partly due to collinearity of those predictors with self-reported literacy. Younger cohorts (Millennials and Gen Z) expressed greater willingness than Boomers, paralleling the pattern observed for self-reported perception of statistical literacy.

We also found a notable association with income. Those reporting incomes between $50k and $150k showing greater willingness to use statistics if they understood it better compared to both those earning less than $50k and those earning more than $150k. Those who identified as politically independent expressed greater willingness to use statistics if they understood it better. Compared to Non-Hispanic White, Black/African American respondents expressed greater willingness to use statistics if they understood it better.

### Informal consistency check: Statistical software use

**Fig 6:**
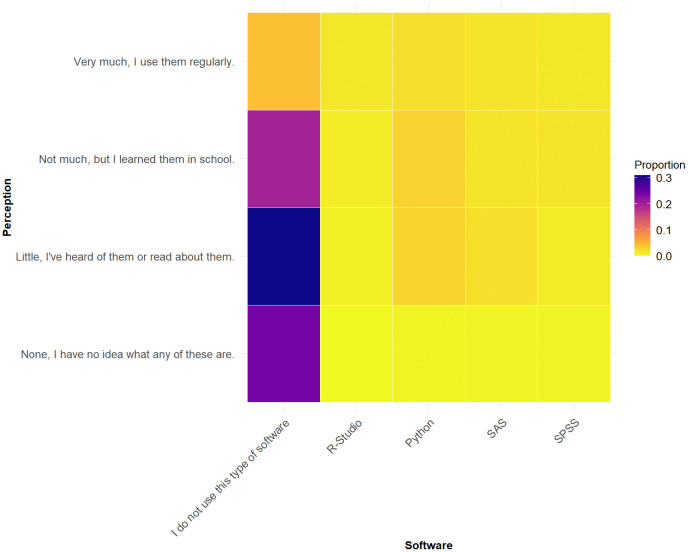
Self-Reported Statistical Literacy by Statistical Software Use.

Respondents were also asked to identify statistical software that they have encountered or used, providing an informal check on consistency with the construct for self-reported perception of statistical literacy. We found that 79% of the respondents did not encounter any of the software included. Fig 6 shows the distribution of responses according to self-reported literacy. Consistently, 96% of those who reported no literacy in statistics also encountered none of the statistical software. Among those who reported learning statistics in school, only 27% reported using at least one of the software packages. Among those who reported using statistics regularly, 60% identified one of the software options, with the remainder likely using tools not listed among the survey options. Cross-tabulation further highlights the alignment between self-reported literacy and actual tool use. Only 5% of those who reported using none of the software were people who also self-reported using statistics regularly, while those who self-reported having no statistical literacy were also about 5% of those who reported using any of the statistical tools.

## Discussion

Our finding that the majority of U.S. adults perceive themselves as having little to no working knowledge of statistics aligns closely with results from large-scale assessments of objective numeracy skills. The PIAAC reports that only 12% of U.S. adults attain a level of numeracy that encompasses explicit components of statistical literacy [24]; given that the PIAAC numeracy levels are composites of multiple numeracy elements, it is reasonable to infer that fewer still are competent at using data under uncertainty for statistically sound decisions. Our data, while measuring perceived rather than actual proficiency, reflect a strikingly similar distribution, suggesting that self-assessments may track differences in educational exposure and skill development. Furthermore, because perception of competence tends to overestimate actual competence [39], and misuse of statistics among those who use it is well-documented [40], the 10.8% who report being regular users of statistics likely overstates the proportion who apply statistics soundly in practice.

The finding that approximately 90% of U.S. adults expressed at least some willingness to use statistics in decision-making—conditional on improved understanding—points to an important but underutilized source of public motivation. A strong foundation in statistical literacy is widely recognized as critical to engaging with published information across scientific fields [41,42], yet the National Science Foundation has reported that while American public trust in science remains high, engagement with scientific information remains low [43]. Our results suggest that deficits in perceived statistical literacy may be a meaningful contributor to this gap: adults appear willing to engage with quantitative evidence if they feel sufficiently equipped to do so. While technical expertise in conducting statistical analysis is not required for engagement with published research, the ability to interpret statistical findings and evaluate the credibility of conclusions drawn ‌‌from them is essential [44].

The modest positive association between perceived statistical literacy and conditional willingness (Spearman Rho = 0.36) is consistent with the broader self-efficacy literature, which shows that perceived competence predicts engagement and persistence in a given domain [45–47]. The fact that even among those reporting no statistical knowledge, 78% expressed some conditional willingness suggests that motivational barriers may be lower than skill barriers. Interventions aimed at improving both actual and perceived statistical competence could therefore have outsized effects on public use of statistics.

The strong positive associations of higher educational attainment and younger generational cohort with self-reported statistical literacy are consistent with the progressive integration of statistics and data literacy into K–12 and higher education curricula over at least the past two decades [10,20]. The finding that Gen Z reports both the lowest proportion with no statistical knowledge and the highest proportion of regular users reinforces this trend and further suggests that younger adults’ more frequent encounters with quantitative content in digital and media environments may also contribute to greater familiarity with statistical concepts. The persistence of education and generation effects on willingness to use statistics, even after accounting for self-reported literacy, likely reflects the collinearity of these variables rather than independent pathways.

The slightly higher self-reported statistical literacy among males may reflect documented disparities in access to advanced mathematics and statistics coursework, differences in quantitative self-efficacy, or gendered occupational pathways [48], but this may also reflect documented gender gap in self-assessment of mathematics ability in general [49].

The income gradient in willingness, with those in the $50k–$150k range expressing greater willingness than both lower- and higher-income groups, may reflect differential access to higher education and quantitatively oriented occupations. The greater willingness among politically independent respondents and Black/African American respondents, relative to their respective reference groups, are novel findings that merit further investigation; possible explanations include different patterns of exposure to contested statistical claims in policy or health contexts, though the cross-sectional and descriptive nature of the current data preclude causal inference.

The cross-tabulation of self-reported statistical literacy with statistical software use provides informal convergent validation of the literacy construct. The strong correspondence between self-reported literacy levels and software encounter rates, from 96% non-encounter among those reporting no literacy to 60% encounter among regular users, suggests that the single-item literacy measure captures meaningful variation in quantitative experience, notwithstanding the limitations of self-report acknowledged below.

## Conclusion and limitations

Using a nationally representative survey of the U.S. adults, we find that perceived statistical literacy remains low, with only a small minority reporting regular use of statistics. At the same time, most adults expressed at least moderate willingness to use statistics in decision-making, provided they had a better understanding of statistical concepts. We also found that self-reported literacy was strongly associated with both higher level of educational attainment and younger generational cohorts, while willingness to use statistics in decision-making was strongly linked to individual’s perceived literacy. These findings highlight that perceived knowledge is associated with openness to applying statistics in decision-making.

This study has several important limitations. First, the central constructs are framed as single‑item questions that only measure respondents’ self-reported perceptions about statistical literacy and how much they claim they are willing to make decisions based on statistics. We do not measure actual statistical literacy nor the actual extent to which respondents would apply such literacy if it was present. Also, the wording of the statistical literacy item (“statistics and p-values”) may narrow the construct toward familiarity with formal statistical tools for decision-making which we explicitly acknowledge. As prior research has shown, perception of competence is not necessarily correlated with actual competence [39,50,51] which certainly applies to statistical literacy especially given the many misconceptions about statistical practice that even those who use statistics regularly fall into [40,52]. Accordingly, our results should not be interpreted as evidence of actual statistical proficiency or effective statistical reasoning, but rather as indicators of perceived understanding and conditional willingness to engage with statistics.

Second, even in this context of measuring only self-reported perception of statistical literacy, we recognize that statistical literacy is widely understood as multidimensional, involving the interpretation, critique, and communication of statistical information, as well as openness to trusting that information, and that our construction focuses only on perception/recollection of exposure to and use of statistical concepts. We were limited by parameters set by the survey provider on how long the questions can be and framed our questions as best we could within such limitations. Third, this study is mainly descriptive and exploratory. Because our results reflect only hypothetical willingness to use statistics and is explicitly conditioned on self-reported understanding, the observed associations should be interpreted as descriptive of attitudes rather than as evidence of independent behavioral effects.

Our findings should be interpreted alongside evidence from large-scale assessments of quantitative and data-related skills. Results from PIAAC and PISA consistently show that a substantial proportion of U.S. adults and students demonstrate limited proficiency in numeracy, data interpretation, and reasoning under uncertainty [25,26]. Although these assessments measure objective skills rather than self-perceptions, the distribution of performance levels observed in PIAAC and in the PISA uncertainty and data subscale closely mirrors the distribution of perceived statistical understanding reported in this study. This alignment suggests that respondents’ self-assessments may reflect underlying educational exposure and skill development rather than purely subjective confidence. Taken together, our findings and those from PIAAC and PISA highlight both persistent skill gaps and a readiness to engage more deeply with statistical information.

The results indicate that many U.S. adults are aware of limitations in their statistical understanding and express greater willingness to use statistics if they felt better equipped to do so. This combination of recognized need and latent motivation underscores an important opportunity for education policy and practice. This is especially relevant for AI‑related learning as contemporary AI systems rely heavily on statistical reasoning. Strengthening statistical literacy may therefore serve as a practical entry point for helping the public learn to interpret and use AI‑generated information responsibly. This should extend beyond higher education and STEM-focused pathways to include K–12 curricula and adult continuing education, positioning statistical literacy as a core civic skill in an increasingly data- and AI-driven society. Future research should integrate objective and subjective measures to better understand how perceived and actual statistical competence jointly shape public engagement with statistical information.

## Supporting information

S1 File Verasight JSM 2025 Omnibus Survey. (PDF)
