# Supplementary material for: Self-reported perception of statistical literacy: Evidence from a National Survey of U.S. Adults
Source: PLoS One. 2026 Jun 24;21(6):e0350282. doi: 10.1371/journal.pone.0350282 (PMC13293397; doi:10.1371/journal.pone.0350282)
Supplement: S1 File — (PDF) [file pone.0350282.s001.pdf]

2025-091 JSM Omnibus

Start of Block: Intro

Intro We’d like to ask you some questions from multiple contributors interested in a wide range of topics. There are no right or wrong answers. You may have answered questions like these before. We are interested in your answers today, whether they have changed or are the same.

End of Block: Intro

Start of Block: Module A

A_1 Are you familiar with very low carbohydrate diets, such as a Keto or carnivore diet?

- Yes
- No
- Unsure

Page Break

A_2 What were your primary motivations for hand-washing during the pandemic? *Select all that apply*.

- [ ] Fear of COVID-19 infection
- [ ] Public health advice
- [ ] Social expectations
- [ ] Personal hygiene habits
- [ ] Workplace or school policies
- [ ] ⊗I wasn’t motivated by any specific reasons to hand-wash during the pandemic

Page Break

X

A_3 A cancer biomarker test looks for biological signs of cancer in your body. What is the highest price you are willing to pay for a 95% reliable cancer biomarker test which gives you personalized treatment options?

- ○ Less than $2,500
- ○ $2,500 - $5,000
- ○ $5,001 - $10,000
- ○ $10,001 - $20,000
- ○ More than $20,000 if necessary

Page Break

A_4 If you had to guess, how would you describe the rate of teenage pregnancies in the US?

- ○ Relatively low
- ○ Relatively high
- ○ About average

Page Break

A_5 If you experience your mental health getting worse, what would you do first to address it?

- ○ Seek help from a healthcare provider
- ○ Take a federally approved medication
- ○ Take a drug other than a federally approved medication
- ○ Seek help from family and friends
- ○ Do nothing, expecting my problems to go away on their own

Page Break

A_6 How lonely are you?

- Not at all lonely
- Slightly lonely
- A little lonely
- Moderately lonely
- Quite lonely
- Very lonely
- Extremely lonely

Page Break

A_7 To what extent do you agree or disagree with the following statement? As a child, I could count on my parent for emotional support.

- Completely disagree
- Disagree
- Somewhat disagree
- Neither agree nor disagree
- Somewhat agree
- Agree
- Completely agree

Page Break

A_8 To what extent do you agree or disagree with the following statement? My parent didn't seem to notice if I was upset or struggling.

- Completely disagree
- Disagree
- Somewhat disagree
- Neither agree nor disagree
- Somewhat agree
- Agree
- Completely agree

Page Break

A_9 To what extent do you agree or disagree with the following statement? Even when I was a teenager, my parent treated me like a little kid who couldn't be trusted.

- Completely Disagree
- Disagree
- Somewhat Disagree
- Neither Agree nor Disagree
- Somewhat Agree
- Agree
- Completely Agree

Page Break

A_10 To what extent do you agree or disagree with the following statement? My parent was close with their parents before I was born.

- Completely disagree
- Disagree
- Somewhat disagree
- Neither agree nor disagree
- Somewhat agree
- Agree
- Completely agree

Page Break

A_11 Did you have a pet growing up?

- Yes
- No

Page Break

A_12 How often do you consume alcohol?

- Never
- Very rarely
- Rarely
- Occasionally
- Regularly
- Frequently
- Very frequently

Page Break

A_13 Do you consider vaping an e-cigarette to be smoking?

- ◯ Yes
- ◯ No

Page Break

A_14 Do you believe in climate change?

- ○ Yes
- ○ No
- ○ Unsure

Page Break

A_15 Over the next 4 years, do you think U.S. relations with other countries will become:

- ○ Stronger than they are now
- ○ Weaker than they are now
- ○ About the same

Page Break

A_16 To what extent do you agree or disagree with the following statement? A spiritual awakening/revival is needed to address the high levels of uncertainty worldwide.

- Strongly Agree
- Agree
- Neutral
- Disagree
- Strongly Disagree

Page Break

A_17 How much do you trust AI (Large Language Models such as ChatGPT, Gemini, and Grok) when it comes to its recommendations for lifestyle changes to improve your overall health?

- Not at all
- Slightly
- Moderately
- Very
- Extremely
- Prefer not to answer

Page Break

A_18 Which of the following best describes how you think AI impacts student learning?

- ○ Personalizes learning
- ○ Reduces deep thinking
- ○ Acts as a study aid
- ○ Adds convenience, not depth
- ○ Encourages tech overuse
- ○ Promotes dependancy
- ○ Impact is still unclear

Page Break

A_19 To what extent do you agree or disagree with the following statement? I believe statistics is useful in everyday life.

- Strongly Disagree
- Disagree
- Neutral
- Agree
- Strongly Agree

Page Break

A_20 Where have you recently encountered statistics in your daily life? *Select all that apply.*

- ☐ News reports
- ☐ Social media
- ☐ Advertising
- ☐ School/work
- ☐ Medical/health information
- ☐ Political discussions
- ☐ ⊗I haven't noticed statistics used recently

Page Break

A_21 How much do you understand about statistics and p-values?

- None, I have no idea what any of these are.
- Little, I've heard of them or read about them.
- Not much, but I learned them in school.
- Very much, I use them regularly.

Page Break

A_22 How often would you base decisions on reported statistics if you understood it better?

- Always
- Often
- Sometimes
- Never

Page Break

A_23 Which of the following statistical softwares, if any, have you used most?

- ○ I do not use this type of software
- ○ R-Studio
- ○ Python
- ○ SAS
- ○ SPSS

Page Break

A_24 We would like to know what you think about how fair or unfair the following gross income would be for you in your current job.   If you were paid **$62,192** for your current job, would you consider that to be:

- ◯ Extremely Unfair Underpayment
- ◯ Very Unfair Underpayment
- ◯ Somewhat Unfair Underpayment
- ◯ Slightly Unfair Underpayment
- ◯ Fair Payment
- ◯ Slightly Unfair Overpayment
- ◯ Somewhat Unfair Overpayment
- ◯ Very Unfair Overpayment
- ◯ Extremely Unfair Overpayment
- ◯ I am not employed

End of Block: Module A
