# Supplementary material for: Self-reported perception of statistical literacy: Evidence from a National Survey of U.S. Adults
Source: PLoS One. 2026 Jun 24;21(6):e0350282. doi: 10.1371/journal.pone.0350282 (PMC13293397; doi:10.1371/journal.pone.0350282)
Supplement: Attachment — Submitted filename: responses_2_03272026_auresp_3.docx [file pone.0350282.s004.docx]

We thank you for your effort and expertise in reviewing our manuscript thoroughly. We address each of the points you raised in this correspondence. We marked our responses in red font.

Major Concerns
Introduction: The introduction does not yet clearly articulate the motivation for analyzing subjective numeracy in this sample alongside participants' hypothetical willingness to apply statistics if qualified. I do not question the relevance of the topic, but the rationale needs to be made more explicit. You note that measurement attempts have been limited (l. 61), yet the survey relies on self-report, which does not directly resolve that gap. You therefore need to develop a more substantive argument for why a self-report approach is appropriate and valuable here.

Response: We have included a discussion on self-reported subjective numeracy and situated our paper as a nationally representative measurement of this, which is our main contribution. We also reordered our objectives to reflect this, as examining associations with demographic variables is just a secondary objective.

Additionally, the introduction lacks elaboration on the broader context of hypothetical willingness to apply statistics. What does the literature say about preferences for data-based or inductive reasoning, and about how people prefer to engage with statistics? Are there pedagogical studies examining outcomes following successful statistical training?

Response: We have addressed this in the revision, specifically exploring the following studies that establish the idea that people who believe they understand mathematics are more likely to use quantitative reasoning. This serves as the motivation for us to also want to measure conditional willingness in our study.

Peters E. Subjective Numeracy and Knowing What You Know. In: Peters E, editor. Innumeracy in the Wild: Misunderstanding and Misusing Numbers [Internet]. Oxford University Press; 2020 [cited 2026 Mar 16]. p. 0. Available from: https://doi.org/10.1093/oso/9780190861094.003.0014 doi:10.1093/oso/9780190861094.003.0014

Peters E, Shoots-Reinhard B. Numeracy and the Motivational Mind: The Power of Numeric Self-efficacy. Med Decis Mak Int J Soc Med Decis Mak. 2022 Aug;42(6):729–40. doi:10.1177/0272989X221099904 PubMed PMID: 35583117.

Finally, given your sample characteristics and analytic approach, the introduction should more explicitly justify why examining demographic factors is a central aim of this study.

Response: We would like to clarify that examining demographic factors is not a central aim. It is only a secondary aim and we have clarified this in the revised introduction and the abstract.

Methods: As a secondary data analysis, this manuscript is subject to specific reporting standards that are not yet fully met. The reader needs considerably more information about the sample. Was the study conducted entirely online? Across all device types? How many individuals were in the sampling pool, and how many were eligible, invited, responded, and completed the survey? Response and completion rates are critical for any claims about representativeness. Please also clarify the weighting method used and specify to which variable the reported margin of error refers. The ethics statement should be included in the Methods section, as it is important for readers to understand what type of consent participants provided during the original primary data collection conducted by the survey company. Structure of the Methods section: Please clearly separate the following subsections: Sample, Design, Measures, Procedure, and Analysis. Within the Procedure subsection, it should be transparent what occurred prior to this particular assessment — for instance, was this part of a multi-topic survey?

Within the Measures subsection, please cite the origin of the items used, or at minimum reference related prior or similar instrument-assessments. Include a description of the software item. Please also add the original questionnaire to the supplementary material.

Response: We have revised the methodology section to address these and included a copy of the survey as part of supplementary material.

Results and Discussion: Please fully disentangle the Results and Discussion sections. As currently written, it is difficult to distinguish your empirical findings from interpretations drawn from the literature. The two sections should be clearly separated so readers can readily identify your original contribution.

Response: We have done this. Thank you.

Sample representativeness: If the sample is representative of the U.S. adult online population without further selection criteria, Table 1 and related population characteristics should be moved to supplementary material, paired with publicly available national statistics that allow readers to assess how well the sample reflects the population along the reported dimensions.

Response: We prefer to retain Table 1 because it provides essential descriptive context about the survey respondents and helps readers understand the composition of the sample before interpreting the main results.

Tables 4, 5, and 6: Please convert these tables into figures. The focus here is on patterns rather than absolute values, and visual representations will communicate these patterns more effectively.

Response: We have done this.

Minor Concerns
• Title: Please reconsider the title. The concept of "conditional willingness" is not immediately clear to the reader, and the title should signal that this is a secondary analysis rather than a prospectively planned survey study.

Response: We have revised the title to remove conditional willingness. We still include it in the manuscript where we now provide a more explicit definition. We do clarify that this is a prospectively planned survey study. We contributed questions to the JSM 2025 Omnibus Survey that Verasight then carried out. All the people who contributed questions to the survey were then provided with the survey results after data collection concluded. These details are now added to the methods section.

• Abstract: The abstract should be rewritten to provide more explicit methodological information — in particular, clearly identifying this as a secondary data analysis, describing the sample, and specifying the study type. Please remove p-values from the abstract. When "conditional willingness" is first mentioned, briefly define it. Associated factors should also be introduced before results are reported.

Response: We have done this.

• References 7 and 8: Please replace these with more recent and methodologically stronger evidence to support the associated claim.

Response: Done

• First paragraph: Please close with a clear statement of what this study aims to contribute.

Response: Done

• Second paragraph: Please consider citing Hoekstra et al. (2014), Psychonomic Bulletin & Review, 21, 1157–1164, and Gigerenzer (2004), The Journal of Socio-Economics, 33(5), 587–606.

Response: Done

• Page numbers: Please add page numbers throughout.

Response: Done

• Decimal formatting: Please report all proportions and confidence intervals to two decimal places.

Response: Done

• Table formatting: Please ensure all tables are consistently formatted according to APA 7 / PLOS ONE guidelines.

Response: Done

• Gender disparity: Please consider discussing the well-documented male tendency toward self-overestimation in the context of your gender-related findings.

Response: Done

• Figure captions: Please revise all figure captions for clarity; several are currently difficult to interpret.

Response: Done

• Generation definitions: Please specify how generational categories were defined.
Response: Done

• Lines 313 and 317: Please correct the errors identified on these lines.
Response: Kindly clarify, we did not see any information on what these errors are.

• Openness to statistics: Please consider whether and how openness to statistics may have influenced perceived statistical literacy, and whether this warrants discussion.
Response: We have included aspects of this before in our limitations but have now made it more explicit.

• Lines 336–338: If education is included as a covariate in the model, it cannot simultaneously serve as part of the explanatory narrative in the discussion. Please clarify this apparent inconsistency.

Response: As the model is exploratory, education was included as a covariate to account for its association with willingness to use statistics. We do acknowledge that this is correlated with self-reported literacy (Lines 361-363)

We look forward to receiving your revised manuscript.

Sincerely
Felix Rebitschek

**Reviewer #2:** I think the authors have done an admirable job in addressing virtually all of the comments and concerns raised in my review, and in Rev#1 as well. The response letter is detailed and clear, and I followed all fixes with much interest, and understand the reactions where authors decided to stay on their original course. I like the expanded theoretical introduction and additional background about PIAAC and PISA, streamlined presentation of the Results, and expanded the Conclusions, with an explicit attention to limitation. Congratulations.

I only have a few remaining comments, the first a bit deeper, the others technical (just changing/adding a couple words):

1. The opening of the paper (i.e., first full paragraph in Introduction) goes heavy into AI issues and explains why statistical literacy matters in this regard – but the AI angle is almost forgotten later, and is only hinted in one word in the closing paragraph of Conclusions. I think that it would be good to close the loop, given the importance of the connection between statistical literacy and AI. Please explain in the closing paragraph in a couple sentences the relevance of the findings to *learning* AI-related stuff. Perhaps - reposition (or paraphrase) a bit of the opening text about AI and at the end, or add brief text along the lines of (informal illustrative text follows): while tentative, the results suggest that at least some of the US public has a basis or readiness related to understanding and using more statistics, hopefully including AI-related statistical ideas. I hope this helps.

Response: We have added this, thank you.

2. In section “Results”: The second main subheader is “Measurement of main constructs”. I think this is misleading - the ‘measurement’ itself (in the sense of the items/scales used) is already covered earlier under “constructs and statistical analysis”. I suggest revising this subheader to something more fitting such as “Findings regarding the main constructs”.

Response: We have done this.

3. In section “Conclusions”: Some quirks in choice of language and internal bridging that can be easily fixed.
(A) in 3rd full paragraph (“Second, even in…”) - see line 7 (line 399 in PDF). Clarify what is “The second outcome” – the referent is unclear, and you already have “Second” twice in this paragraph and the one before it. I suggest to simply clarify what outcome you refer to.

Response: We have done this.

(B) last paragraph of Discussion “Finally,…”): The word “finally” is utterly confusing because it looks like a continuation of listing limitations 9as indicated by “First” and “Second” in prior paragraph. Please replace to something like “In closing/summary” or similar which would indicate to readers you are now switching and going beyond the limitations to the final conclusions.

Response: We have done this.

(C) The closing paragraph is also hefty and mixes various points - I suggest splitting in two, both for readability, and to bring out the separate points it contains and explicate your contributions (and this also serves the suggestion to say a bit more about AI, see #1 above).

Response: We have done this.

7. PLOS authors have the option to publish the peer review history of their article ([what does this mean?](https://nam10.safelinks.protection.outlook.com/?url=https%3A%2F%2Ftrack.editorialmanager.com%2FCL0%2Fhttps%3A%252F%252Fjournals.plos.org%252Fplosone%252Fs%252Feditorial-and-peer-review-process%2523loc-peer-review-history%2F1%2F010f019cf6380036-08336769-a4ee-43dc-bb6a-8116c40038d1-000000%2F56ImEdnKo0q5Idc8cW5ZT_G1Bg3ajGx5vSI6yBVxzsg%3D254&data=05%7C02%7Cmlr6219%40psu.edu%7C353a56069b41474ebf4c08de8347e3d2%7C7cf48d453ddb4389a9c1c115526eb52e%7C0%7C0%7C639092541963995317%7CUnknown%7CTWFpbGZsb3d8eyJFbXB0eU1hcGkiOnRydWUsIlYiOiIwLjAuMDAwMCIsIlAiOiJXaW4zMiIsIkFOIjoiTWFpbCIsIldUIjoyfQ%3D%3D%7C0%7C%7C%7C&sdata=dVr2PK8j3RGMYj%2BRz4L2PQWG2kEoGfJLQhYnrBx7x1o%3D&reserved=0)). If published, this will include your full peer review and any attached files.


If you choose “no”, your identity will remain anonymous but your review may still be made public.


**Do you want your identity to be public for this peer review?** For information about this choice, including consent withdrawal, please see our [Privacy Policy](https://nam10.safelinks.protection.outlook.com/?url=https%3A%2F%2Ftrack.editorialmanager.com%2FCL0%2Fhttps%3A%252F%252Fwww.plos.org%252Fprivacy-policy%2F1%2F010f019cf6380036-08336769-a4ee-43dc-bb6a-8116c40038d1-000000%2FakxD4h9YIRnZlHTTiusgTojuh_WjoJFX9rCM28TPumw%3D254&data=05%7C02%7Cmlr6219%40psu.edu%7C353a56069b41474ebf4c08de8347e3d2%7C7cf48d453ddb4389a9c1c115526eb52e%7C0%7C0%7C639092541964005190%7CUnknown%7CTWFpbGZsb3d8eyJFbXB0eU1hcGkiOnRydWUsIlYiOiIwLjAuMDAwMCIsIlAiOiJXaW4zMiIsIkFOIjoiTWFpbCIsIldUIjoyfQ%3D%3D%7C0%7C%7C%7C&sdata=8UVd1AInQRgb61UlI9lcX4ZNPWT1Fskxdyegxzl%2FjRQ%3D&reserved=0).

Reviewer #1: No

Reviewer #2: **Yes:** Iddo Gal

[NOTE: If reviewer comments were submitted as an attachment file, they will be attached to this email and accessible via the submission site. Please log into your account, locate the manuscript record, and check for the action link "View Attachments". If this link does not appear, there are no attachment files.]

To ensure your figures meet our technical requirements, please review our figure guidelines: <https://journals.plos.org/plosone/s/figures>

You may also use PLOS’s free figure tool, NAAS, to help you prepare publication quality figures: <https://journals.plos.org/plosone/s/figures#loc-tools-for-figure-preparation>.

NAAS will assess whether your figures meet our technical requirements by comparing each figure against our figure specifications.
