# Supplementary material for: Self-reported perception of statistical literacy: Evidence from a National Survey of U.S. Adults
Source: PLoS One. 2026 Jun 24;21(6):e0350282. doi: 10.1371/journal.pone.0350282 (PMC13293397; doi:10.1371/journal.pone.0350282)
Supplement: Attachment — Submitted filename: Response to Reviewers.docx [file pone.0350282.s002.docx]

Dear Reviewers,

We thank you for your effort and expertise in reviewing our manuscript thoroughly. We address each of the points you raised in this correspondence. We marked our responses in red font.

**REVIEWER 1**

A first general strength is that the topic is timely, and the introduction highlights convincingly why statistical literacy matters. The dataset is also solid. The survey weighting, sample design, and the use of survey-weighted ordinal logistic regression are all appropriate. The modelling procedures are technically sound, and the authors check the proportional odds assumption and consider alternative specifications when needed. From a purely statistical perspective, the analysis has been carried out with a good level of rigor.
However, the main concern of my review is the way the central constructs are conceptualized and measured. The manuscript repeatedly refers to “statistical literacy”, but the measure used in the survey is a single item that does not reflect the multidimensional nature of statistical literacy. Established definitions, such as the one by Gal (2002), cited in the text, describe statistical literacy as the ability to interpret, critique, and communicate statistical information. In contrast, the survey item focuses on whether respondents understand “statistics and p-values”. This phrasing mixes different aspects, general familiarity, school exposure, and the use of formal statistical tools, and it places a particular emphasis on p-values, which are a rather technical concept. The inclusion of p-values may unintentionally narrow the meaning of the construct and may exclude forms of everyday statistical reasoning that are important for civic decision-making. For this reason, the results should not be interpreted as measuring statistical literacy in a broad sense, but rather as measuring self-reported familiarity or exposure to formal statistics. I would encourage the authors to address this more directly in the manuscript and to frame their conclusions accordingly.

Response: We agree with the reviewer and emphasize that the construct measures self-reported familiarity and perceived exposure to formal statistical concepts. In framing this construct, we did mean for it to incorporate the aspects the reviewer identified, which we intended to represent reasonable ordinal levels of perceived literacy. To address this limitation more directly, we have revised the manuscript in several ways. First, we now consistently refer to the construct as “self-reported perception of statistical literacy”, and we explicitly distinguish it from objective or comprehensive measures of statistical literacy. Second, we have added clarifying language in the Methods section acknowledging that the construct emphasizes familiarity with formal statistical concepts. Third, we have revised the Results and Conclusion & Limitation sections to frame our findings accordingly, avoiding claims that extend beyond what the measure can support.

A similar issue arises with the second outcome variable, which asks how often respondents would base decisions on statistics “if they understood it better”. This is a hypothetical question that reflects attitudes or intentions, not actual behavior. It is also directly conditioned on the first item, which makes the two variables conceptually intertwined. This structure increases the risk that the statistical associations between the two outcomes are overstated simply because of how the items are formulated. It would help the manuscript if the authors were to acknowledge these limitations explicitly and be more cautious in interpreting the strength and meaning of the associations.

Response: We agree with the reviewer’s assessment. We now acknowledge that the second outcome captures hypothetical intentions rather than observed behavior and that its conditional phrasing makes it conceptually related to the self-reported literacy measure. To address this, we have added clarifying language in the Methods, Discussion, and Limitations sections. We emphasize that our study is descriptive and exploratory, and its value is mainly in the capture of the perspectives of a U.S.-population representative sample about the constructs of interest.

Although the statistical analysis itself is well implemented, the measurement issues affect how far the results can be taken. Several key claims in the manuscript, especially those that generalize about “statistical literacy in the U.S. adult population”, go beyond what can be concluded from single-item measures of this kind. The same applies to the broader implications for AI literacy and public trust. These are important themes, but they require a clearer link between the empirical measures used in the study and the conceptual claims made in the introduction and conclusion.
Because of these issues, I would recommend revising the manuscript so that the discussion is better aligned with what is actually measured. This includes describing more carefully what the survey items capture (and what they do not capture), explaining the limitations that come with single-item measures, and being more reserved in drawing broader conclusions. A somewhat expanded limitations section would also help clarify the scope of the findings.

Response: We agree with these inputs and have revised the conclusion with an expanded explanation of the study’s limitations.

There are a few more minor points. The link between the software-use question and the main construct should also be presented as a rough consistency check rather than a form of validation. Given that self-report measures are sensitive to overconfidence and to gender differences in self-assessment, a short reflection on this might also strengthen the discussion.

Response: We revised this accordingly and added the concerns of known overconfidence and gender differences in self-reporting in the limitations.

In summary, the manuscript is based on valuable data and uses appropriate analytical methods. However, the central constructs are not measured in a way that fully matches the conceptual framing in the paper, and this affects the interpretation of the results. I therefore recommend major revisions. Once these conceptual and interpretive issues are addressed more directly, the paper could make a useful contribution to ongoing discussions about data literacy, AI literacy, and public understanding of statistics.

Response: We deeply appreciate the reviewer’s time and expertise in thoroughly reviewing our work. We have revised the manuscript to clarify the distinction between the conceptual framing and the constructs measured, explicitly acknowledge measurement limitations, and adjust the interpretation and conclusions accordingly. We believe these changes address the reviewer’s concerns and strengthen the paper’s contribution.

**REVIEWER 2**

Reviewer #2: This paper reports on results from a national survey of US adults, focusing on two new core questions regarding perceived statistical knowledge and willingness to use statistics in decisions, the relationship between them, and how responses are influenced by various bio-data and other correlates. The data source appears very adequate, and the authors certainly know their craft and pack sophisticated results.

Overall, I like the paper and believe that it presents unique findings, which are much needed and has the potential to add a new perspective and inform extant discussions regarding the development of statistical literacy and data literacy. That said, the paper needs much further work to fulfill its potential. For now, the results are the main part of the paper (and appear to be written for statisticians), and the opening and closing are relatively slim. The paper requires a heavy revision to enhance its contribution to knowledge and to a much needed dialogue regarding the future of statistics and data literacy education and skill development of the adult population. It needs a further conceptualization of the problem, a streamlined and more accessible presentation of the results, and a deeper Discussion. These issues are elaborated below, with suggestions aiming to help you improve the paper in several ways.

1. INTRODUCTION: The scholarly background is too brief. The current text sketches some relevant background, and quickly focuses on the absence of credible information about statistical skills in the population. I fully agree that we need more such info - but you should elaborate on the actual skill levels related to two arenas - adult numeracy (adults), and mathematical literacy or mathematics (K-12) - which are part of the overall knowledge scene and relevant to the current problem.

Let me first sketch these two, then explain why: Regarding adults: you cite two sources related to PIAAC (i.e., the OECD survey of adult skills), but you just list them, never say anything about the actual national skill levels among adults (which were just published in Dec 2024). For K-12: you ignore school-level sources altogether! FYI, this involves PISA but also NAEP - and PISA 2022 had a statistics subscale!. (And, you could also refer to findings related to functional areas in which adults actually make decisions, such as health literacy surveys).
The above are critical omissions, if you intend to develop later any argument, as you do, related to the need to improve school-level education in data literacy, because these statistics show the challenge involved – and because they are needed to interpret your findings. The proportions at levels 1, 2, 3 etc both in PIAAC *and* in PISA roughly match the percentages you report in the paper for the four levels of perceived statistical literacy. Thus, PIAAC and PISA results provide a strong alternative explanation for your findings, which you do not acknowledge. The upshot of the above – please expand the Introduction and the background about actual skill levels. This will also help your Discussion (see later).

Response: We thank the reviewer for highlighting these important omissions and providing the excellent resources. In response, we have substantially expanded the Introduction Section to situate our study within the broader empirical landscape of adult numeracy and school-based mathematical literacy. Specifically, we now summarize recent findings from PIAAC (including the December 2024 release) to describe national adult skill levels, and we incorporate evidence from PISA 2022, particularly the uncertainty and data subscale to contextualize K–12 preparation. We explicitly discuss how these large-scale assessments provide an alternative and complementary explanation for the distribution of perceived statistical literacy observed in our data, and we draw on these comparisons in the Discussion to more carefully interpret our findings and their implications for education and adult learning.

2. Your Introduction should also open up on the fact you are measuring *perceived (self-reported)* skills or actions - these are not the actual skill levels. Readers should be forewarned upfront of this conceptual distinction, which should also be revisited in the Discussion (see below). FYI, there is ample literature on “subjective numeracy” and how it differs and its *low* correlations with “objective numeracy” – this should be easy to find via a simple search, a lot of it from the medical/health literacy field, and from the ‘human judgment and decision making’ field - which is especially relevant given the nature of your second core variable.

Response: We agree with the reviewer and have revised the manuscript to foreground from the outset that the study examines perceived (self-reported) statistical understanding and willingness, not objective skill levels. This distinction is now made explicit in the Introduction, title, and throughout the manuscript, and it is revisited in the Discussion. We have also included existing studies on self-reported statistical literacy in the U.S., which focus mainly on students in higher education rather than the general population, and use this to explain our contribution to literature as a nationally representative survey on self-reported perception of statistical literacy and willingness to use statistics for decision-making. We also cite sources on associations between objective vs subjective competence in the limitations. We do note though that we are aware of the low or even negative correlation, especially in the context of psychological theories like Dunning-Kruger, and it is not the point of our paper to imply that people who have high self-perception about their statistical literacy are also able to use statistics in decision-making properly, only that they would be more likely to use statistics if they believed they understood it better.

3. RESULTS: The results contain a lot of interesting and important information. However, the writing is complex and assumes a high level of statistical sophistication, in particular the extensive use of odds-ratios to model associations and influences of covariates. I of course realize that this is PLOS ONE and it is seemingly geared for scientists, but restructuring and some simplification are needed to improve effective scientific communication - especially if you want to inform the dialogue among educational or policy communities and expect them to pay attention. The points below elaborate and provide suggestions.

Response: We thank the reviewer for the suggestions provided in subsequent comments, we have revised the Results section to streamline the presentation, reduce technical complexity where possible, and improve clarity and accessibility for a broader interdisciplinary audience.

4. The overall narrative in the Results takes readers through displays (Tables, figures), which in several places appear to define subsections – but the internal structure is unclear, and details abound. Please lead readers in a more consistent manner through your analytic questions, use subtitles to organize, explain upfront what you are after and why, and then refer to the tables and figures (but see later ideas about trimming).

Response: We agree with this suggestion and have included subsections to make the structure clearer. We have also included introductory statements at the start of each subsection stating what the reader should expect.

5. The Results highlight the central relationship between your two core variables. i.e., self-reported statistical literacy and willingness to use statistics in decisions. However, we are talking about the association between two ordinal variables with only four levels, so why complicated odds ratios? please start with simply presenting a 2-way table between these 4-level variables, with row percents and/or column percents, and walk readers through the interpretation of the patterns. (You already employ a similar 4-level structure in Tables 2, 3 and 4). Further, consider presenting a simple correlational statistic for ordinal variables – the level of association between the two most important variables should be made accessible to all readers (even a humble Spearman or Kendall may do for this). The combination of this association + the trends in the cells enable you to sketch a rich story. Odds ratios can come later, to the extent needed, followed by the important covariational analysis.

Response: We agree with the reviewer’s suggestion. Aligned with the previous comment, our main results are now presented in the “Measurement of Main Constructs” subsection. The 2-way table that the reviewer is describing is actually Table 5, and we realize that this should have been more prominently included. Thus, we have moved this to the main results subjection as well (it is now table 3) and we added the spearman correlation measurement in this subsection as well.

6. More about odds ratios (first discussed on p.15 of the PDF): These are notoriously confusing to understand, especially when dealing with ordinal variables (not for continuous variables) and given that they are on a log scale. To illustrate the complexity of your narrative when you try to explain what was found, you write: “The intercepts of the model show progressively higher odds thresholds for each fixed ordinal level, indicating smaller differences in the latent trait between those who report “None” or “Little,” – this is circular and overly abstract technical jargon (you refer to higher odds ratios when explaining odds ratios).
I think that the bulk of your core findings can be presented without so many odds ratios (see also next point about the long tables with odds ratios results). When you start on odds ratios, please explain what are they and what the values mean, both regarding the information in the text, and in the Figures that show them (there are several). Then, connect this to the meaning of the changing odds values in terms of modeling the response variables, and how all this connects with your research question - which is the core of why you do all this.

Response: We understand the perspective of the reviewer but maintain that the use of ordinal regression is the simplest, systematic way to present the constructs in the context of covariates. We believe that these covariates are typically of interest to readers and so including them in the model is informative. However at the same time, we recognize that some readers may not be interested in them at all and want to focus more on the core findings, which is why we had grouped those together in the “Measurement of Main Constructs” subsection and then created separate subsections for where the ordinal regression models appear. This way, readers can easily navigate to which results they are interested in. Regarding the particular statement about odds ratios mentioned for the intercepts, that is standard language for ordinal regression when describing how difficult/easy it is to jump from a lower category to the next higher category. We have also added introductory explanation and example on how to interpret the odds ratios in the results while discussing Figure 1.

7. Too many tables and Figures: I think that a couple of tables or figures can be combined or trimmed (and instead summarized in the text) – this will help you communicate your key patterns in a simpler way, and clear space for a stronger Discussion. For example, I question the need for the very long Figure 1 and Figure 2. They look appealing, but the bottom half is repetitive (no differences between subgroups), and you already summarize this in the text: “None of the other demographic variables (…) were found to have statistically significant association with self-reported statistical literacy”. Better spend the space on explaining the meaning of the values, with a couple examples, e.g., for where there is a large difference between subgroups, or no difference.

Response: We understand this point of view. However, we would like to keep the figures as they are so that readers can see the entirety of the models. The study itself is descriptive and exploratory, and so readers may be interested to see the estimates and confidence intervals regardless of whether they were statistically significant.

8. Clarity regarding Table 3 and 4: These are briefly explained (see p.15) but (a) it is unclear what are the values in the tables (not explained in the text), and the text itself is confusing since you refer within a single paragraph both to odds-ratios and to proportions, and throw in percentages, and you state that “Complete cross tabulations are in Table 4” but it is unclear what is shown in table 4.

Response: We apologize for the confusion. Our intention was to examine the associations found via odds ratios in Figure 1 more closely by looking into the crosstabulation of our main construct with those variables. This avoids needing to interpret odds ratios directly (which as the reviewer pointed out, may not be of interest to some readers) by translating them into more familiar crosstabulation language. This way, we maintain the display of odds ratios for those who wish to see them and are comfortable with interpreting them as odds ratios, while providing consistent interpretation of those odds ratios but in less regression-specific technical language. We have rephrased this subsection to reflect this intention better.

9. DISCUSSION. This section is underwhelming. It is devoid of any linkages to scholarly literature (no references) and omits major points that have to be discussed in order to provide an effective scholarly discussion and to push the applied conclusions.

9.1. Please improve the interpretation of the findings: What can you conclude from the statistics you show, when it is well known (see my point #2) that the correlation between perceived and actual skills is relatively low? Note this is not a methodological limitation but a conceptual pillar which may undermine your conclusions, so needs attention.
Likewise, discuss the possibility that the distributions of your two core variables (i.e., the proportions in each of the four levels) are affected by people’s numeracy, or self-perceived numeracy, not by how well they may understand statistics. This alternative interpretation is more likely when you refer to adult populations that are vulnerable in terms of their numeracy, such as those with lower education / income / elderly (you do have covariation analyses about them).

Response: We agree with the reviewer on the need for more discussion and apologize for this shortcoming. We have revised the work to provide more robust discussion of presented results in the context of existing literature.

9.2. Add a Limitations subsection. There are several issues, the most basic of which is that your core variables “How much do you understand about statistics and p-values?” suffers from a classic “double-barrel” problem (we teach to avoid it in questionnaire design). This phrasing can distort the measurement, since most people know about statistics (e.g., even from sports) but not about p-values. It is surprising that the polling agency (Verasight) let that slip through.

Response: We have added limitations in our conclusion section and renamed the section accordingly. Regarding the wording of the question, we understand the perspective of the reviewer however, we clarify that it emphasizes familiarity with formal statistical concepts (about which p-values is one of the most recognizable terms) as typically taught for decision-making, rather than everyday numerical reasoning. We also believe that the structuring of our responses help with better contextualization of the question. We have added this in our limitations.

9.3. Further develop the conclusions and recommendations so they are more specific – for now you speak about the need to improve understanding of data literacy to help with AI – but what about statistical literacy for the sake of improving people’s statistical literacy (and helping citizen empowerment)? Or improved understanding and decisions regarding health issues?
Also, rethink and possibly specify who your target audiences are - since there are multiple arenas of action and delivery systems: adult education, statistics and data-science at the school (K-12) level, and post-secondary (college) level. Each of these teaches about statistics, and deserve separate acknowledgment.

Response: We have revised the Conclusion to provide more specific implications grounded in our results, emphasizing statistical literacy as a civic skill beyond AI contexts. We now explicitly distinguish implications for K–12 education, adult continuing education, and postsecondary instruction, clarifying how each arena relates to the observed gaps in perceived statistical understanding and willingness to use statistics.


9.4. Please say something about implications of your work (or of the limitations) for needed future research.

Response: We have added a clear discussion of future research directions, including the need for multidimensional and performance-based measures of statistical literacy, longitudinal designs linking perceived and objective competence, and studies examining how perceived literacy shapes engagement with statistical and AI-mediated information in applied contexts such as health and public decision-making.

10. In closing: My comments do not aim to criticize, but to show ways in which the paper can be improved, and how to strengthen its impact. Good luck!

Response: We deeply appreciate the reviewer’s time and expertise in thoroughly reviewing our work and hope that the changes we implemented are able to address their concerns.
