# Supplementary material for: Self-reported perception of statistical literacy: Evidence from a National Survey of U.S. Adults
Source: PLoS One. 2026 Jun 24;21(6):e0350282. doi: 10.1371/journal.pone.0350282 (PMC13293397; doi:10.1371/journal.pone.0350282)
Supplement: Peer review history

# Peer review: Decision Letter 0

**DOI:** 10.1371/journal.pone.0350282.r001

**Authors:** Felix Rebitschek

7 Jan 2026

PONE-D-25-53057

Self-Reported Statistical Literacy and Conditional Willingness to Use Statistics in Everyday Decision-Making: Evidence from a National Survey of U.S. Adults

PLOS One

Dear Dr. Ramos,

Thank you for submitting your manuscript to PLOS ONE. After careful consideration, we feel that it has merit but does not fully meet PLOS ONE’s publication criteria as it currently stands. Therefore, we invite you to submit a revised version of the manuscript that addresses the points raised during the review process.

Please submit your revised manuscript by Feb 21 2026 11:59PM. If you will need more time than this to complete your revisions, please reply to this message or contact the journal office at plosone@plos.org. When you're ready to submit your revision, log on to https://www.editorialmanager.com/pone/ and select the 'Submissions Needing Revision' folder to locate your manuscript file.

Please include the following items when submitting your revised manuscript:

- A letter that responds to each point raised by the academic editor and reviewer(s). You should upload this letter as a separate file labeled 'Response to Reviewers'.
- A marked-up copy of your manuscript that highlights changes made to the original version. You should upload this as a separate file labeled 'Revised Manuscript with Track Changes'.
- An unmarked version of your revised paper without tracked changes. You should upload this as a separate file labeled 'Manuscript'.

If you would like to make changes to your financial disclosure, please include your updated statement in your cover letter. Guidelines for resubmitting your figure files are available below the reviewer comments at the end of this letter.

If applicable, we recommend that you deposit your laboratory protocols in protocols.io to enhance the reproducibility of your results. Protocols.io assigns your protocol its own identifier (DOI) so that it can be cited independently in the future. For instructions see: https://journals.plos.org/plosone/s/submission-guidelines#loc-laboratory-protocols. Additionally, PLOS ONE offers an option for publishing peer-reviewed Lab Protocol articles, which describe protocols hosted on protocols.io. Read more information on sharing protocols at https://plos.org/protocols?utm_medium=editorial-email&utm_source=authorletters&utm_campaign=protocols.

We look forward to receiving your revised manuscript.

Kind regards,

Felix G. Rebitschek

Academic Editor

PLOS One

Journal Requirements:

When submitting your revision, we need you to address these additional requirements.

1. Please ensure that your manuscript meets PLOS ONE's style requirements, including those for file naming. The PLOS ONE style templates can be found at

https://journals.plos.org/plosone/s/file?id=wjVg/PLOSOne_formatting_sample_main_body.pdf and

https://journals.plos.org/plosone/s/file?id=ba62/PLOSOne_formatting_sample_title_authors_affiliations.pdf

2. For studies involving third-party data, we encourage authors to share any data specific to their analyses that they can legally distribute. PLOS recognizes, however, that authors may be using third-party data they do not have the rights to share. When third-party data cannot be publicly shared, authors must provide all information necessary for interested researchers to apply to gain access to the data. (https://journals.plos.org/plosone/s/data-availability#loc-acceptable-data-access-restrictions)

For any third-party data that the authors cannot legally distribute, they should include the following information in their Data Availability Statement upon submission:

1) A description of the data set and the third-party source

2) If applicable, verification of permission to use the data set

3) Confirmation of whether the authors received any special privileges in accessing the data that other researchers would not have

4) All necessary contact information others would need to apply to gain access to the data

3. If the reviewer comments include a recommendation to cite specific previously published works, please review and evaluate these publications to determine whether they are relevant and should be cited. There is no requirement to cite these works unless the editor has indicated otherwise.

**Additional Editor Comments:**

Dear Authors,

Thank you for submitting your work!

You have received very constructive reviews that help you address some major besides minor issues. Please examine the reviews' facets thoroughly to improve your manuscript for the revision.

Sincerely,

Felix Rebitschek

[Note: HTML markup is below. Please do not edit.]

Reviewers' comments:

Reviewer's Responses to Questions

**Comments to the Author**

1. Is the manuscript technically sound, and do the data support the conclusions?

The manuscript must describe a technically sound piece of scientific research with data that supports the conclusions. Experiments must have been conducted rigorously, with appropriate controls, replication, and sample sizes. The conclusions must be drawn appropriately based on the data presented.

Reviewer #1: Partly

Reviewer #2: Partly

**********

2. Has the statistical analysis been performed appropriately and rigorously?

Reviewer #1: Yes

Reviewer #2: Yes

**********

3. Have the authors made all data underlying the findings in their manuscript fully available?

The PLOS Data policy requires authors to make all data underlying the findings described in their manuscript fully available without restriction, with rare exception (please refer to the Data Availability Statement in the manuscript PDF file). The data should be provided as part of the manuscript or its supporting information, or deposited to a public repository. For example, in addition to summary statistics, the data points behind means, medians and variance measures should be available. If there are restrictions on publicly sharing data—e.g. participant privacy or use of data from a third party—those must be specified.

Reviewer #1: No

Reviewer #2: Yes

**********

4. Is the manuscript presented in an intelligible fashion and written in standard English?

PLOS ONE does not copyedit accepted manuscripts, so the language in submitted articles must be clear, correct, and unambiguous. Any typographical or grammatical errors should be corrected at revision, so please note any specific errors here.

Reviewer #1: Yes

Reviewer #2: Yes

**********

5. Review Comments to the Author

Please use the space provided to explain your answers to the questions above. You may also include additional comments for the author, including concerns about dual publication, research ethics, or publication ethics. (Please upload your review as an attachment if it exceeds 20,000 characters)

Reviewer #1: The paper deals with a very relevant topic, namely the role of statistical literacy in a data-driven society and its connection to public understanding of AI systems. The authors make use of a nationally representative survey, and the statistical analyses themselves are conducted with care. The study has clear potential, and I believe it can be publishable after substantial revisions. My comments below are intended to help strengthen the conceptual clarity and the interpretation of the results.

A first general strength is that the topic is timely, and the introduction highlights convincingly why statistical literacy matters. The dataset is also solid. The survey weighting, sample design, and the use of survey-weighted ordinal logistic regression are all appropriate. The modelling procedures are technically sound, and the authors check the proportional odds assumption and consider alternative specifications when needed. From a purely statistical perspective, the analysis has been carried out with a good level of rigor.

However, the main concern of my review is the way the central constructs are conceptualized and measured. The manuscript repeatedly refers to “statistical literacy”, but the measure used in the survey is a single item that does not reflect the multidimensional nature of statistical literacy. Established definitions, such as the one by Gal (2002), cited in the text, describe statistical literacy as the ability to interpret, critique, and communicate statistical information. In contrast, the survey item focuses on whether respondents understand “statistics and p-values”. This phrasing mixes different aspects, general familiarity, school exposure, and the use of formal statistical tools, and it places a particular emphasis on p-values, which are a rather technical concept. The inclusion of p-values may unintentionally narrow the meaning of the construct and may exclude forms of everyday statistical reasoning that are important for civic decision-making. For this reason, the results should not be interpreted as measuring statistical literacy in a broad sense, but rather as measuring self-reported familiarity or exposure to formal statistics. I would encourage the authors to address this more directly in the manuscript and to frame their conclusions accordingly.

A similar issue arises with the second outcome variable, which asks how often respondents would base decisions on statistics “if they understood it better”. This is a hypothetical question that reflects attitudes or intentions, not actual behavior. It is also directly conditioned on the first item, which makes the two variables conceptually intertwined. This structure increases the risk that the statistical associations between the two outcomes are overstated simply because of how the items are formulated. It would help the manuscript if the authors were to acknowledge these limitations explicitly and be more cautious in interpreting the strength and meaning of the associations.

Although the statistical analysis itself is well implemented, the measurement issues affect how far the results can be taken. Several key claims in the manuscript, especially those that generalize about “statistical literacy in the U.S. adult population”, go beyond what can be concluded from single-item measures of this kind. The same applies to the broader implications for AI literacy and public trust. These are important themes, but they require a clearer link between the empirical measures used in the study and the conceptual claims made in the introduction and conclusion.

Because of these issues, I would recommend revising the manuscript so that the discussion is better aligned with what is actually measured. This includes describing more carefully what the survey items capture (and what they do not capture), explaining the limitations that come with single-item measures, and being more reserved in drawing broader conclusions. A somewhat expanded limitations section would also help clarify the scope of the findings.

There are a few more minor points. The link between the software-use question and the main construct should also be presented as a rough consistency check rather than a form of validation. Given that self-report measures are sensitive to overconfidence and to gender differences in self-assessment, a short reflection on this might also strengthen the discussion.

In summary, the manuscript is based on valuable data and uses appropriate analytical methods. However, the central constructs are not measured in a way that fully matches the conceptual framing in the paper, and this affects the interpretation of the results. I therefore recommend major revisions. Once these conceptual and interpretive issues are addressed more directly, the paper could make a useful contribution to ongoing discussions about data literacy, AI literacy, and public understanding of statistics.

Reviewer #2: This paper reports on results from a national survey of US adults, focusing on two new core questions regarding perceived statistical knowledge and willingness to use statistics in decisions, the relationship between them, and how responses are influenced by various bio-data and other correlates. The data source appears very adequate, and the authors certainly know their craft and pack sophisticated results.

Overall, I like the paper and believe that it presents unique findings, which are much needed and has the potential to add a new perspective and inform extant discussions regarding the development of statistical literacy and data literacy. That said, the paper needs much further work to fulfill its potential. For now, the results are the main part of the paper (and appear to be written for statisticians), and the opening and closing are relatively slim. The paper requires a heavy revision to enhance its contribution to knowledge and to a much needed dialogue regarding the future of statistics and data literacy education and skill development of the adult population. It needs a further conceptualization of the problem, a streamlined and more accessible presentation of the results, and a deeper Discussion. These issues are elaborated below, with suggestions aiming to help you improve the paper in several ways.

1. INTRODUCTION: The scholarly background is too brief. The current text sketches some relevant background, and quickly focuses on the absence of credible information about statistical skills in the population. I fully agree that we need more such info - but you should elaborate on the actual skill levels related to two arenas - adult numeracy (adults), and mathematical literacy or mathematics (K-12) - which are part of the overall knowledge scene and relevant to the current problem.

Let me first sketch these two, then explain why: Regarding adults: you cite two sources related to PIAAC (i.e., the OECD survey of adult skills), but you just list them, never say anything about the actual national skill levels among adults (which were just published in Dec 2024). For K-12: you ignore school-level sources altogether! FYI, this involves PISA but also NAEP - and PISA 2022 had a statistics subscale!. (And, you could also refer to findings related to functional areas in which adults actually make decisions, such as health literacy surveys).

The above are critical omissions, if you intend to develop later any argument, as you do, related to the need to improve school-level education in data literacy, because these statistics show the challenge involved – and because they are needed to interpret your findings. The proportions at levels 1, 2, 3 etc both in PIAAC *and* in PISA roughly match the percentages you report in the paper for the four levels of perceived statistical literacy. Thus, PIAAC and PISA results provide a strong alternative explanation for your findings, which you do not acknowledge. The upshot of the above – please expand the Introduction and the background about actual skill levels. This will also help your Discussion (see later).

2. Your Introduction should also open up on the fact you are measuring *perceived (self-reported)* skills or actions - these are not the actual skill levels. Readers should be forewarned upfront of this conceptual distinction, which should also be revisited in the Discussion (see below). FYI, there is ample literature on “subjective numeracy” and how it differs and its *low* correlations with “objective numeracy” – this should be easy to find via a simple search, a lot of it from the medical/health literacy field, and from the ‘human judgment and decision making’ field - which is especially relevant given the nature of your second core variable.

3. RESULTS: The results contain a lot of interesting and important information. However, the writing is complex and assumes a high level of statistical sophistication, in particular the extensive use of odds-ratios to model associations and influences of covariates. I of course realize that this is PLOS ONE and it is seemingly geared for scientists, but restructuring and some simplification are needed to improve effective scientific communication - especially if you want to inform the dialogue among educational or policy communities and expect them to pay attention. The points below elaborate and provide suggestions.

4. The overall narrative in the Results takes readers through displays (Tables, figures), which in several places appear to define subsections – but the internal structure is unclear, and details abound. Please lead readers in a more consistent manner through your analytic questions, use subtitles to organize, explain upfront what you are after and why, and then refer to the tables and figures (but see later ideas about trimming).

5. The Results highlight the central relationship between your two core variables. i.e., self-reported statistical literacy and willingness to use statistics in decisions. However, we are talking about the association between two ordinal variables with only four levels, so why complicated odds ratios? please start with simply presenting a 2-way table between these 4-level variables, with row percents and/or column percents, and walk readers through the interpretation of the patterns. (You already employ a similar 4-level structure in Tables 2, 3 and 4). Further, consider presenting a simple correlational statistic for ordinal variables – the level of association between the two most important variables should be made accessible to all readers (even a humble Spearman or Kendall may do for this). The combination of this association + the trends in the cells enable you to sketch a rich story. Odds ratios can come later, to the extent needed, followed by the important covariational analysis.

6. More about odds ratios (first discussed on p.15 of the PDF): These are notoriously confusing to understand, especially when dealing with ordinal variables (not for continuous variables) and given that they are on a log scale. To illustrate the complexity of your narrative when you try to explain what was found, you write: “The intercepts of the model show progressively higher odds thresholds for each fixed ordinal level, indicating smaller differences in the latent trait between those who report “None” or “Little,” – this is circular and overly abstract technical jargon (you refer to higher odds ratios when explaining odds ratios).

I think that the bulk of your core findings can be presented without so many odds ratios (see also next point about the long tables with odds ratios results). When you start on odds ratios, please explain what are they and what the values mean, both regarding the information in the text, and in the Figures that show them (there are several). Then, connect this to the meaning of the changing odds values in terms of modeling the response variables, and how all this connects with your research question - which is the core of why you do all this.

7. Too many tables and Figures: I think that a couple of tables or figures can be combined or trimmed (and instead summarized in the text) – this will help you communicate your key patterns in a simpler way, and clear space for a stronger Discussion. For example, I question the need for the very long Figure 1 and Figure 2. They look appealing, but the bottom half is repetitive (no differences between subgroups), and you already summarize this in the text: “None of the other demographic variables (…) were found to have statistically significant association with self-reported statistical literacy”. Better spend the space on explaining the meaning of the values, with a couple examples, e.g., for where there is a large difference between subgroups, or no difference.

8. Clarity regarding Table 3 and 4: These are briefly explained (see p.15) but (a) it is unclear what are the values in the tables (not explained in the text), and the text itself is confusing since you refer within a single paragraph both to odds-ratios and to proportions, and throw in percentages, and you state that “Complete cross tabulations are in Table 4” but it is unclear what is shown in table 4.

9. DISCUSSION. This section is underwhelming. It is devoid of any linkages to scholarly literature (no references) and omits major points that have to be discussed in order to provide an effective scholarly discussion and to push the applied conclusions.

9.1. Please improve the interpretation of the findings: What can you conclude from the statistics you show, when it is well known (see my point #2) that the correlation between perceived and actual skills is relatively low? Note this is not a methodological limitation but a conceptual pillar which may undermine your conclusions, so needs attention.

Likewise, discuss the possibility that the distributions of your two core variables (i.e., the proportions in each of the four levels) are affected by people’s numeracy, or self-perceived numeracy, not by how well they may understand statistics. This alternative interpretation is more likely when you refer to adult populations that are vulnerable in terms of their numeracy, such as those with lower education / income / elderly (you do have covariation analyses about them).

9.2. Add a Limitations subsection. There are several issues, the most basic of which is that your core variables “How much do you understand about statistics and p-values?” suffers from a classic “double-barrel” problem (we teach to avoid it in questionnaire design). This phrasing can distort the measurement, since most people know about statistics (e.g., even from sports) but not about p-values. It is surprising that the polling agency (Verasight) let that slip through.

9.3. Further develop the conclusions and recommendations so they are more specific – for now you speak about the need to improve understanding of data literacy to help with AI – but what about statistical literacy for the sake of improving people’s statistical literacy (and helping citizen empowerment)? Or improved understanding and decisions regarding health issues?

Also, rethink and possibly specify who your target audiences are - since there are multiple arenas of action and delivery systems: adult education, statistics and data-science at the school (K-12) level, and post-secondary (college) level. Each of these teaches about statistics, and deserve separate acknowledgment.

9.4. Please say something about implications of your work (or of the limitations) for needed future research.

10. In closing: My comments do not aim to criticize, but to show ways in which the paper can be improved, and how to strengthen its impact. Good luck!

**********

6. PLOS authors have the option to publish the peer review history of their article (what does this mean?). If published, this will include your full peer review and any attached files.

If you choose “no”, your identity will remain anonymous but your review may still be made public.

**Do you want your identity to be public for this peer review?** For information about this choice, including consent withdrawal, please see our Privacy Policy.

Reviewer #1: **Yes:** Florian Weber-Stein

Reviewer #2: No

**********

[NOTE: If reviewer comments were submitted as an attachment file, they will be attached to this email and accessible via the submission site. Please log into your account, locate the manuscript record, and check for the action link "View Attachments". If this link does not appear, there are no attachment files.]

To ensure your figures meet our technical requirements, please review our figure guidelines: https://journals.plos.org/plosone/s/figures

You may also use PLOS’s free figure tool, NAAS, to help you prepare publication quality figures: https://journals.plos.org/plosone/s/figures#loc-tools-for-figure-preparation.

NAAS will assess whether your figures meet our technical requirements by comparing each figure against our figure specifications.

# Author comment: Author response to Decision Letter 1

**DOI:** 10.1371/journal.pone.0350282.r002

29 Jan 2026

Responses were included as a separate word file with filename "Response to Reviewers"

Attachment Submitted filename: Response to Reviewers.docx

# Peer review: Decision Letter 1

**DOI:** 10.1371/journal.pone.0350282.r003

**Authors:** Felix Rebitschek

16 Mar 2026

PONE-D-25-53057R1

Self-Reported Perception of Statistical Literacy and Conditional Willingness to Use Statistics in Everyday Decision-Making: Evidence from a National Survey of U.S. Adults

PLOS One

Dear Dr. Ramos,

Thank you for submitting your manuscript to PLOS ONE. After careful consideration, we feel that it has merit but does not fully meet PLOS ONE’s publication criteria as it currently stands. Therefore, we invite you to submit a revised version of the manuscript that addresses the points raised during the review process.

Please submit your revised manuscript by Apr 30 2026 11:59PM. If you will need more time than this to complete your revisions, please reply to this message or contact the journal office at plosone@plos.org. When you're ready to submit your revision, log on to https://www.editorialmanager.com/pone/ and select the 'Submissions Needing Revision' folder to locate your manuscript file.

Please include the following items when submitting your revised manuscript:

- A letter that responds to each point raised by the academic editor and reviewer(s). You should upload this letter as a separate file labeled 'Response to Reviewers'.
- A marked-up copy of your manuscript that highlights changes made to the original version. You should upload this as a separate file labeled 'Revised Manuscript with Track Changes'.
- An unmarked version of your revised paper without tracked changes. You should upload this as a separate file labeled 'Manuscript'.

-->If you would like to make changes to your financial disclosure, please include your updated statement in your cover letter. Guidelines for resubmitting your figure files are available below the reviewer comments at the end of this letter.

If applicable, we recommend that you deposit your laboratory protocols in protocols.io to enhance the reproducibility of your results. Protocols.io assigns your protocol its own identifier (DOI) so that it can be cited independently in the future. For instructions see: https://journals.plos.org/plosone/s/submission-guidelines#loc-laboratory-protocols. Additionally, PLOS ONE offers an option for publishing peer-reviewed Lab Protocol articles, which describe protocols hosted on protocols.io. Read more information on sharing protocols at https://plos.org/protocols?utm_medium=editorial-email&utm_source=authorletters&utm_campaign=protocols.

We look forward to receiving your revised manuscript.

Kind regards,

Felix G. Rebitschek

Academic Editor

PLOS One

**Journal Requirements:**

1. If the reviewer comments include a recommendation to cite specific previously published works, please review and evaluate these publications to determine whether they are relevant and should be cited. There is no requirement to cite these works unless the editor has indicated otherwise.

2. Please review your reference list to ensure that it is complete and correct. If you have cited papers that have been retracted, please include the rationale for doing so in the manuscript text, or remove these references and replace them with relevant current references. Any changes to the reference list should be mentioned in the rebuttal letter that accompanies your revised manuscript. If you need to cite a retracted article, indicate the article’s retracted status in the References list and also include a citation and full reference for the retraction notice.

**Additional Editor Comments:**

Dear Authors,

Thank you for revising your manuscript to address the reviewers' concerns. After careful consideration, however, a few issues remain that require further attention before the manuscript can be accepted. I outline these below.

Major Concerns

Introduction: The introduction does not yet clearly articulate the motivation for analyzing subjective numeracy in this sample alongside participants' hypothetical willingness to apply statistics if qualified. I do not question the relevance of the topic, but the rationale needs to be made more explicit. You note that measurement attempts have been limited (l. 61), yet the survey relies on self-report, which does not directly resolve that gap. You therefore need to develop a more substantive argument for why a self-report approach is appropriate and valuable here. Additionally, the introduction lacks elaboration on the broader context of hypothetical willingness to apply statistics. What does the literature say about preferences for data-based or inductive reasoning, and about how people prefer to engage with statistics? Are there pedagogical studies examining outcomes following successful statistical training? Finally, given your sample characteristics and analytic approach, the introduction should more explicitly justify why examining demographic factors is a central aim of this study.

Methods: As a secondary data analysis, this manuscript is subject to specific reporting standards that are not yet fully met. The reader needs considerably more information about the sample. Was the study conducted entirely online? Across all device types? How many individuals were in the sampling pool, and how many were eligible, invited, responded, and completed the survey? Response and completion rates are critical for any claims about representativeness. Please also clarify the weighting method used and specify to which variable the reported margin of error refers. The ethics statement should be included in the Methods section, as it is important for readers to understand what type of consent participants provided during the original primary data collection conducted by the survey company.

Structure of the Methods section: Please clearly separate the following subsections: Sample, Design, Measures, Procedure, and Analysis. Within the Procedure subsection, it should be transparent what occurred prior to this particular assessment — for instance, was this part of a multi-topic survey? Within the Measures subsection, please cite the origin of the items used, or at minimum reference related prior or similar instrument-assessments. Include a description of the software item. Please also add the original questionnaire to the supplementary material.

Results and Discussion: Please fully disentangle the Results and Discussion sections. As currently written, it is difficult to distinguish your empirical findings from interpretations drawn from the literature. The two sections should be clearly separated so readers can readily identify your original contribution.

Sample representativeness: If the sample is representative of the U.S. adult online population without further selection criteria, Table 1 and related population characteristics should be moved to supplementary material, paired with publicly available national statistics that allow readers to assess how well the sample reflects the population along the reported dimensions.

Tables 4, 5, and 6: Please convert these tables into figures. The focus here is on patterns rather than absolute values, and visual representations will communicate these patterns more effectively.

Minor Concerns

• Title: Please reconsider the title. The concept of "conditional willingness" is not immediately clear to the reader, and the title should signal that this is a secondary analysis rather than a prospectively planned survey study.

• Abstract: The abstract should be rewritten to provide more explicit methodological information — in particular, clearly identifying this as a secondary data analysis, describing the sample, and specifying the study type. Please remove p-values from the abstract. When "conditional willingness" is first mentioned, briefly define it. Associated factors should also be introduced before results are reported.

• References 7 and 8: Please replace these with more recent and methodologically stronger evidence to support the associated claim.

• First paragraph: Please close with a clear statement of what this study aims to contribute.

• Second paragraph: Please consider citing Hoekstra et al. (2014), Psychonomic Bulletin & Review, 21, 1157–1164, and Gigerenzer (2004), The Journal of Socio-Economics, 33(5), 587–606.

• Page numbers: Please add page numbers throughout.

• Decimal formatting: Please report all proportions and confidence intervals to two decimal places.

• Table formatting: Please ensure all tables are consistently formatted according to APA 7 / PLOS ONE guidelines.

• Gender disparity: Please consider discussing the well-documented male tendency toward self-overestimation in the context of your gender-related findings.

• Figure captions: Please revise all figure captions for clarity; several are currently difficult to interpret.

• Generation definitions: Please specify how generational categories were defined.

• Lines 313 and 317: Please correct the errors identified on these lines.

• Openness to statistics: Please consider whether and how openness to statistics may have influenced perceived statistical literacy, and whether this warrants discussion.

• Lines 336–338: If education is included as a covariate in the model, it cannot simultaneously serve as part of the explanatory narrative in the discussion. Please clarify this apparent inconsistency.

We look forward to receiving your revised manuscript.

Sincerely

Felix Rebitschek

[Note: HTML markup is below. Please do not edit.]

Reviewers' comments:

Reviewer's Responses to Questions

**Comments to the Author**

1. If the authors have adequately addressed your comments raised in a previous round of review and you feel that this manuscript is now acceptable for publication, you may indicate that here to bypass the “Comments to the Author” section, enter your conflict of interest statement in the “Confidential to Editor” section, and submit your "Accept" recommendation.

Reviewer #1: All comments have been addressed

Reviewer #2: (No Response)

**********

2. Is the manuscript technically sound, and do the data support the conclusions?

The manuscript must describe a technically sound piece of scientific research with data that supports the conclusions. Experiments must have been conducted rigorously, with appropriate controls, replication, and sample sizes. The conclusions must be drawn appropriately based on the data presented.

Reviewer #1: Yes

Reviewer #2: Yes

**********

3. Has the statistical analysis been performed appropriately and rigorously?

Reviewer #1: Yes

Reviewer #2: Yes

**********

4. Have the authors made all data underlying the findings in their manuscript fully available?

The PLOS Data policy requires authors to make all data underlying the findings described in their manuscript fully available without restriction, with rare exception (please refer to the Data Availability Statement in the manuscript PDF file). The data should be provided as part of the manuscript or its supporting information, or deposited to a public repository. For example, in addition to summary statistics, the data points behind means, medians and variance measures should be available. If there are restrictions on publicly sharing data—e.g. participant privacy or use of data from a third party—those must be specified.

Reviewer #1: No

Reviewer #2: (No Response)

**********

5. Is the manuscript presented in an intelligible fashion and written in standard English?

PLOS ONE does not copyedit accepted manuscripts, so the language in submitted articles must be clear, correct, and unambiguous. Any typographical or grammatical errors should be corrected at revision, so please note any specific errors here.

Reviewer #1: Yes

Reviewer #2: Yes

**********

6. Review Comments to the Author

Please use the space provided to explain your answers to the questions above. You may also include additional comments for the author, including concerns about dual publication, research ethics, or publication ethics. (Please upload your review as an attachment if it exceeds 20,000 characters)

**Reviewer #1:** Thank you for the opportunity to review the revised version of this manuscript.

I appreciate the authors’ careful and thorough revisions. The manuscript has been substantially improved, particularly with respect to the clarification of the central constructs and the alignment between the conceptual framing and what is empirically measured. The expanded limitations section and the more cautious interpretation of the findings appropriately address the core concerns raised in the previous review.

While the study continues to rely on self-reported, single-item measures—which naturally constrains the strength of the conclusions—the authors now acknowledge these boundaries clearly and consistently. The discussion is more proportionate to the evidence presented, and the contribution is framed in a way that is methodologically transparent.

In its revised form, I believe the manuscript meets the journal’s standards and can be accepted for publication.

**Reviewer #2:** I think the authors have done an admirable job in addressing virtually all of the comments and concerns raised in my review, and in Rev#1 as well. The response letter is detailed and clear, and I followed all fixes with much interest, and understand the reactions where authors decided to stay on their original course. I like the expanded theoretical introduction and additional background about PIAAC and PISA, streamlined presentation of the Results, and expanded the Conclusions, with an explicit attention to limitation. Congratulations.

I only have a few remaining comments, the first a bit deeper, the others technical (just changing/adding a couple words):

1. The opening of the paper (i.e., first full paragraph in Introduction) goes heavy into AI issues and explains why statistical literacy matters in this regard – but the AI angle is almost forgotten later, and is only hinted in one word in the closing paragraph of Conclusions. I think that it would be good to close the loop, given the importance of the connection between statistical literacy and AI. Please explain in the closing paragraph in a couple sentences the relevance of the findings to *learning* AI-related stuff. Perhaps - reposition (or paraphrase) a bit of the opening text about AI and at the end, or add brief text along the lines of (informal illustrative text follows): while tentative, the results suggest that at least some of the US public has a basis or readiness related to understanding and using more statistics, hopefully including AI-related statistical ideas. I hope this helps.

2. In section “Results”: The second main subheader is “Measurement of main constructs”. I think this is misleading - the ‘measurement’ itself (in the sense of the items/scales used) is already covered earlier under “constructs and statistical analysis”. I suggest revising this subheader to something more fitting such as “Findings regarding the main constructs”.

3. In section “Conclusions”: Some quirks in choice of language and internal bridging that can be easily fixed.

(A) in 3rd full paragraph (“Second, even in…”) - see line 7 (line 399 in PDF). Clarify what is “The second outcome” – the referent is unclear, and you already have “Second” twice in this paragraph and the one before it. I suggest to simply clarify what outcome you refer to.

(B) last paragraph of Discussion “Finally,…”): The word “finally” is utterly confusing because it looks like a continuation of listing limitations 9as indicated by “First” and “Second” in prior paragraph. Please replace to something like “In closing/summary” or similar which would indicate to readers you are now switching and going beyond the limitations to the final conclusions.

(C) The closing paragraph is also hefty and mixes various points - I suggest splitting in two, both for readability, and to bring out the separate points it contains and explicate your contributions (and this also serves the suggestion to say a bit more about AI, see #1 above).

**********

7. PLOS authors have the option to publish the peer review history of their article (what does this mean?). If published, this will include your full peer review and any attached files.

If you choose “no”, your identity will remain anonymous but your review may still be made public.

**Do you want your identity to be public for this peer review?** For information about this choice, including consent withdrawal, please see our Privacy Policy.

Reviewer #1: No

Reviewer #2: **Yes:** Iddo Gal

**********

[NOTE: If reviewer comments were submitted as an attachment file, they will be attached to this email and accessible via the submission site. Please log into your account, locate the manuscript record, and check for the action link "View Attachments". If this link does not appear, there are no attachment files.]

To ensure your figures meet our technical requirements, please review our figure guidelines: https://journals.plos.org/plosone/s/figures

You may also use PLOS’s free figure tool, NAAS, to help you prepare publication quality figures: https://journals.plos.org/plosone/s/figures#loc-tools-for-figure-preparation.

NAAS will assess whether your figures meet our technical requirements by comparing each figure against our figure specifications.

# Author comment: Author response to Decision Letter 2

**DOI:** 10.1371/journal.pone.0350282.r004

30 Mar 2026

Responses to reviewers was included as an uploaded file.

Attachment Submitted filename: responses_2_03272026.docx

# Editor report: Decision Letter 2

**DOI:** 10.1371/journal.pone.0350282.r005

**Authors:** Felix Rebitschek

10 Apr 2026

PONE-D-25-53057R2

Self-Reported Perception of Statistical Literacy: Evidence from a National Survey of U.S. Adults

PLOS One

Dear Dr. Ramos,

Thank you for submitting your manuscript to PLOS ONE. After careful consideration, we feel that it has merit but does not fully meet PLOS ONE’s publication criteria as it currently stands. Therefore, we invite you to submit a revised version of the manuscript that addresses the points raised during the review process.

Please submit your revised manuscript by May 25 2026 11:59PM. If you will need more time than this to complete your revisions, please reply to this message or contact the journal office at plosone@plos.org. When you're ready to submit your revision, log on to https://www.editorialmanager.com/pone/ and select the 'Submissions Needing Revision' folder to locate your manuscript file.

Please include the following items when submitting your revised manuscript:

- A letter that responds to each point raised by the academic editor and reviewer(s). You should upload this letter as a separate file labeled 'Response to Reviewers'.
- A marked-up copy of your manuscript that highlights changes made to the original version. You should upload this as a separate file labeled 'Revised Manuscript with Track Changes'.
- An unmarked version of your revised paper without tracked changes. You should upload this as a separate file labeled 'Manuscript'.

If you would like to make changes to your financial disclosure, please include your updated statement in your cover letter. Guidelines for resubmitting your figure files are available below the reviewer comments at the end of this letter.

If applicable, we recommend that you deposit your laboratory protocols in protocols.io to enhance the reproducibility of your results. Protocols.io assigns your protocol its own identifier (DOI) so that it can be cited independently in the future. For instructions see: https://journals.plos.org/plosone/s/submission-guidelines#loc-laboratory-protocols. Additionally, PLOS ONE offers an option for publishing peer-reviewed Lab Protocol articles, which describe protocols hosted on protocols.io. Read more information on sharing protocols at https://plos.org/protocols?utm_medium=editorial-email&utm_source=authorletters&utm_campaign=protocols.

As the corresponding author, your ORCID iD is verified in the submission system and will appear in the published article. PLOS supports the use of ORCID, and we encourage all coauthors to register for an ORCID iD and use it as well. Please encourage your coauthors to verify their ORCID iD within the submission system before final acceptance, as unverified ORCID iDs will not appear in the published article. *Only* the individual author can complete the verification step; PLOS staff *cannot* verify ORCID iDs on behalf of authors.

We look forward to receiving your revised manuscript.

Kind regards,

Felix G. Rebitschek

Academic Editor

PLOS One

Journal Requirements:

If the reviewer comments include a recommendation to cite specific previously published works, please review and evaluate these publications to determine whether they are relevant and should be cited. There is no requirement to cite these works unless the editor has indicated otherwise.

Please review your reference list to ensure that it is complete and correct. If you have cited papers that have been retracted, please include the rationale for doing so in the manuscript text, or remove these references and replace them with relevant current references. Any changes to the reference list should be mentioned in the rebuttal letter that accompanies your revised manuscript. If you need to cite a retracted article, indicate the article’s retracted status in the References list and also include a citation and full reference for the retraction notice.

Additional Editor Comments:

Dear Authors,

Please provide a manuscript version including track changes of the introduction and discussion in accordance to your response to reviewers and editor.

Sincerely

Felix Rebitschek

[Note: HTML markup is below. Please do not edit.]

[NOTE: If reviewer comments were submitted as an attachment file, they will be attached to this email and accessible via the submission site. Please log into your account, locate the manuscript record, and check for the action link "View Attachments". If this link does not appear, there are no attachment files.]

To ensure your figures meet our technical requirements, please review our figure guidelines: https://journals.plos.org/plosone/s/figures

You may also use PLOS’s free figure tool, NAAS, to help you prepare publication quality figures: https://journals.plos.org/plosone/s/figures#loc-tools-for-figure-preparation.

NAAS will assess whether your figures meet our technical requirements by comparing each figure against our figure specifications.

# Author comment: Author response to Decision Letter 3

**DOI:** 10.1371/journal.pone.0350282.r006

13 Apr 2026

We have uploaded the manuscript version with tracked changes. We apologize for the oversight. We previously uploaded this document but after we got another revision request regarding the figures which we have also corrected, we assumed the previous changes had already been reviewed and so uploaded a version with only the latest query about the figures tracked. We have now replaced that file with the previous one we uploaded that correctly contains tracked changes addressing the concerns of the reviewer and the editor.

We assume this was the only concern in the decision letter as we found no other new comments. Thank you.

Attachment Submitted filename: responses_2_03272026_auresp_3.docx

# Editor report: Decision Letter 3

**DOI:** 10.1371/journal.pone.0350282.r007

**Authors:** Felix Rebitschek

12 May 2026

Self-Reported Perception of Statistical Literacy: Evidence from a National Survey of U.S. Adults

PONE-D-25-53057R3

Dear Dr. Ramos,

We’re pleased to inform you that your manuscript has been judged scientifically suitable for publication and will be formally accepted for publication once it meets all outstanding technical requirements.

Within one week, you’ll receive an e-mail detailing the required amendments. When these have been addressed, you’ll receive a formal acceptance letter and your manuscript will be scheduled for publication.

An invoice will be generated when your article is formally accepted. Please note, if your institution has a publishing partnership with PLOS and your article meets the relevant criteria, all or part of your publication costs will be covered. Please make sure your user information is up-to-date by logging into Editorial Manager at Editorial Manager® and clicking the ‘Update My Information' link at the top of the page. For questions related to billing, please contact billing support.

If your institution or institutions have a press office, please notify them about your upcoming paper to help maximize its impact. If they’ll be preparing press materials, please inform our press team as soon as possible -- no later than 48 hours after receiving the formal acceptance. Your manuscript will remain under strict press embargo until 2 pm Eastern Time on the date of publication. For more information, please contact onepress@plos.org.

Kind regards,

Felix G. Rebitschek

Academic Editor

PLOS One

Additional Editor Comments (optional):

Dear Authors,

Thank you very much for submitting your revised version and addressing the issues. If you address the last issues, the manuscript can be accepted:

1. The table editing is still inconsistent, please harmonize the styling of your tables.

2. All figures also need consistent font size of any labels, and this also includes to ensure sufficiently large/readable font sizes.

3. The paragraph under Procedure belongs to Sample. What is still missing, is a brief description of the procedure ("A step-by-step listing in chronological order of what participants did during the study") - I referred to that last time - What happened before and after the two items? How long did it take?

4. Even if you keep Table 1 where it is, it needs to be contrasted with the national statistics of U.S. adults (additional column), so the reader can interpret the representativeness of your sample

5. "examine some aspects of" means explore?

Your work is a secondary data analysis. Please make this clearly comprehensible throughout the manuscript.

Thank you for your submission!

Felix Rebitschek

Reviewers' comments:

# Editor report: Acceptance letter

**DOI:** 10.1371/journal.pone.0350282.r008

**Authors:** Felix Rebitschek

PONE-D-25-53057R3

PLOS One

Dear Dr. Ramos,

I'm pleased to inform you that your manuscript has been deemed suitable for publication in PLOS One. Congratulations! Your manuscript is now being handed over to our production team.

At this stage, our production department will prepare your paper for publication. This includes ensuring the following:

* All references, tables, and figures are properly cited

* All relevant supporting information is included in the manuscript submission,

* There are no issues that prevent the paper from being properly typeset

You will receive further instructions from the production team, including instructions on how to review your proof when it is ready. Please keep in mind that we are working through a large volume of accepted articles, so please give us a few days to review your paper and let you know the next and final steps.

Lastly, if your institution or institutions have a press office, please let them know about your upcoming paper now to help maximize its impact. If they'll be preparing press materials, please inform our press team within the next 48 hours. Your manuscript will remain under strict press embargo until 2 pm Eastern Time on the date of publication. For more information, please contact onepress@plos.org.

You will receive an invoice from PLOS for your publication fee after your manuscript has reached the completed accept phase. If you receive an email requesting payment before acceptance or for any other service, this may be a phishing scheme. Learn how to identify phishing emails and protect your accounts at https://explore.plos.org/phishing.

If we can help with anything else, please email us at customercare@plos.org.

Thank you for submitting your work to PLOS ONE and supporting open access.

Kind regards,

PLOS ONE Editorial Office Staff

on behalf of

Dr. Felix G. Rebitschek

Academic Editor

PLOS One
